# Spatial reasoning via recurrent neural dynamics in mouse retrosplenial cortex

**DOI:** 10.1038/s41593-025-01944-z

**Published:** 2025-06-06

**Authors:** Jakob Voigts, Ingmar Kanitscheider, Nicholas J. Miller, Enrique H. S. Toloza, Jonathan P. Newman, Ila R. Fiete, Mark T. Harnett

**Affiliations:** 1 Department of Brain and Cognitive Sciences, MIT, Cambridge, MA USA; 2 McGovern Institute for Brain Research, MIT, Cambridge, MA USA; 3 HHMI Janelia Research Campus, Ashburn, VA USA; 4 OpenAI, San Francisco, CA USA; 5 Department of Physics, MIT, Cambridge, MA USA; 6 Picower Institute for Learning and Memory, MIT, Cambridge, MA USA; 7 Open Ephys Inc., Atlanta, GA USA

**Keywords:** Dynamical systems, Short-term memory

## Abstract

From visual perception to language, sensory stimuli change their meaning depending on previous experience. Recurrent neural dynamics can interpret stimuli based on externally cued context, but it is unknown whether they can compute and employ internal hypotheses to resolve ambiguities. Here we show that mouse retrosplenial cortex (RSC) can form several hypotheses over time and perform spatial reasoning through recurrent dynamics. In our task, mice navigated using ambiguous landmarks that are identified through their mutual spatial relationship, requiring sequential refinement of hypotheses. Neurons in RSC and in artificial neural networks encoded mixtures of hypotheses, location and sensory information, and were constrained by robust low-dimensional dynamics. RSC encoded hypotheses as locations in activity space with divergent trajectories for identical sensory inputs, enabling their correct interpretation. Our results indicate that interactions between internal hypotheses and external sensory data in recurrent circuits can provide a substrate for complex sequential cognitive reasoning.

## Abstract (web summary)

Using a spatial reasoning task in mice, the authors show that retrosplenial cortex encodes spatial hypotheses with well-behaved recurrent dynamics, which can combine these hypotheses with incoming information to resolve ambiguities.

## Main

External context can change the processing of stimuli through recurrent neural dynamics^1^. In this process, the evolution of neural population activity depends on its own history as well as external inputs^2^, giving context-specific meaning to otherwise ambiguous stimuli^3^. To study how hypotheses can be held in memory and serve as internal signals to compute new information, we developed a task that requires sequential integration of spatially separated ambiguous landmarks^4^. In this task, the information needed to disambiguate the stimuli is not provided externally but must be computed, maintained over time and applied to the stimuli by the brain.

## Results

**Fig. 1:**
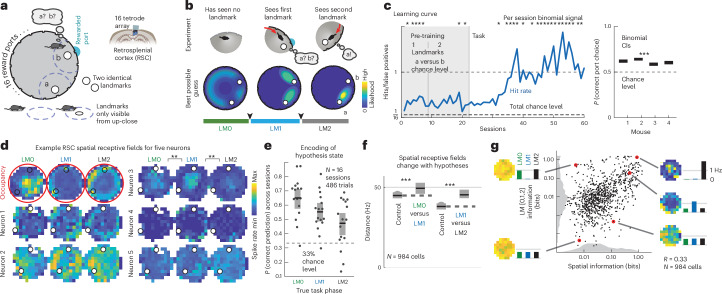
RSC represents spatial information conjunctively with hypothesis states during navigation with locally ambiguous landmarks. **a**, Two perceptually identical landmarks are visible only from close up, and their identity is defined only by their relative location. One of 16 ports, at landmark ‘b,’ delivers reward in response to a nose-poke. The animal must infer which of the two landmarks is ‘b’ to receive reward; wrong pokes result in timeout. Tetrode array recordings in RSC yield 50–90 simultaneous neurons. **b**, Top, schematic example trial; bottom, best possible guesses of the mouse position. LM0, LM1 and LM2 denote task phases when the mouse has seen zero, one or two landmarks and could infer their position with decreasing uncertainty. **c**, Left, example training curve showing *P*_hit_/*P*_false-positive_; random chance level is 1/16 for 16 ports. Mice learned the task at values >1, showing they could disambiguate between the two sequentially visible landmarks. This requires the formation, maintenance and use of spatial hypotheses. Asterisks denote per-session binomial 95% significance for the correct rate. Right, summary statistics show binomial CIs on last half of sessions for all four mice. **d**, Mouse location heatmap from one session (red) with corresponding spatial firing rate profiles for five example cells; color maps are normalized per cell. **e**, Task phase (corresponding to hypothesis states in **b** can be decoded from RSC firing rates. Horizontal line, mean; gray shaded box, 95% CI. **f**, Spatial coding changes between LM1 and LM2 phases (Euclidean distances between spatial firing rate maps, control within versus across condition; see Extended Data Fig. 2a for test by decoding, median and CIs (bootstrap)). **g**, Spatial versus task phase information content of all neurons and position and state encoding for example cells. Gray, sum-normalized histograms (color scale as in **d**).

**Fig. 2:**
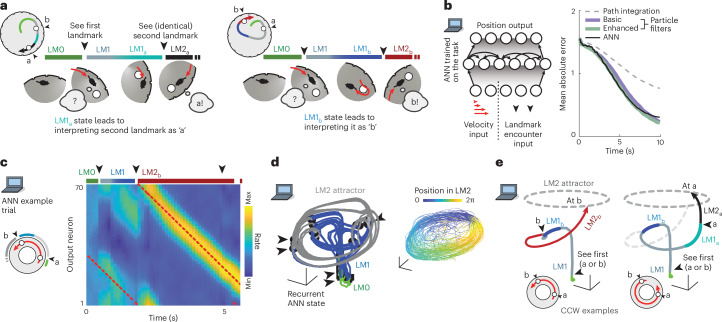
Recurrent neural dynamics can be used to navigate through locally ambiguous landmarks by forming and employing multimodal hypotheses. **a**, Schematic examples of hypothesis-dependent landmark interpretation. Left, mouse encounters first LM, then identifies the second as ‘a’ based on the short relative distance. Right, a different path during LM1 leads the mouse to a different hypothesis state, and to identify the perceptually identical second landmark as ‘b.’ Hypothesis states preceding LM2 are denoted LM1_a_ and LM1_b_, depending on the identity of the second landmark. **b**, Structure of an ANN trained on the task. Inputs encode velocity and landmarks. Right, mean absolute localization error averaged across test trials for random trajectories. **c**, Activity of output neurons ordered by preferred location shows transition between LM0, LM1 and LM2 phases. Red, true location. During LM1 (when the agent has only seen one landmark), two hypotheses are maintained, with convergence to a stable unimodal location estimate in LM2 after encountering the second landmark. **d**, 3D projection from PCA of ANN hidden neuron activities. During LM2, angular position in neural state space reflects position estimate encoding. **e**, Example ANN trajectories for two trials show how identical visual input (black arrowheads) leads the activity to travel to different locations on the LM2 attractor because of different preceding LM1_a/b_ states.

**Extended Data Fig. 1:**
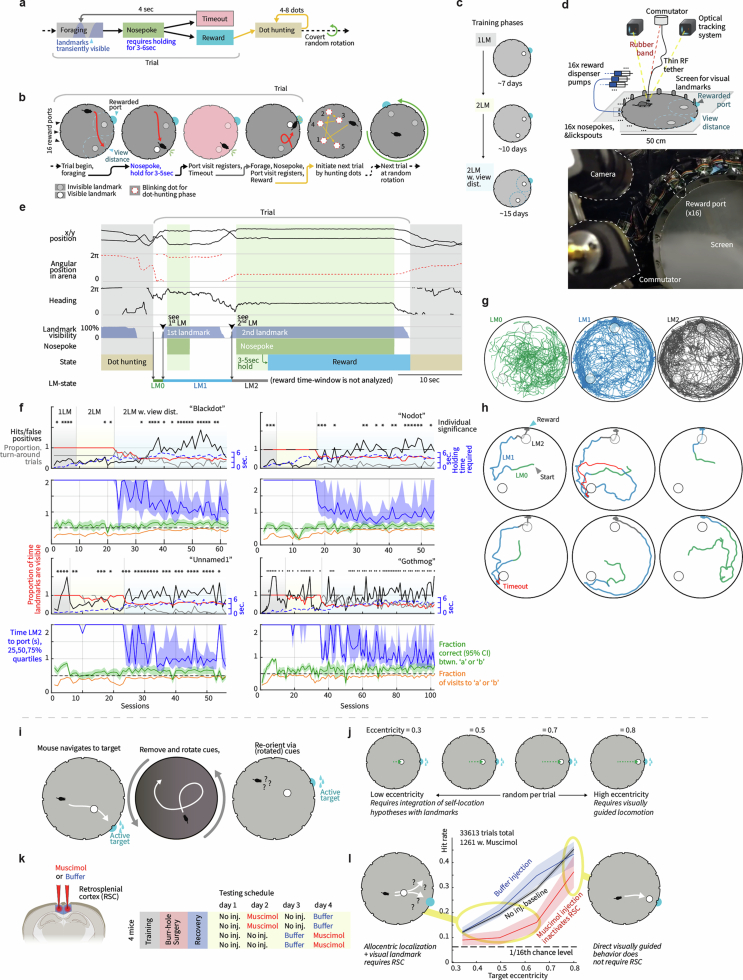
Task structure and behavioral data, and necessity of RSC for egocentric-allocentric computations. (**a**) Schematic of task structure and timing. (**b**) Example trial schematic showing all possible task states (see Methods for more details). Landmarks were formed by white dots displayed on a screen which served as the floor of the arena. They were only made visible when mice crossed a distance threshold. Only one landmark was visible at a time in the final training stage. Nose-pokes were only registered after mice held their nose in the port for a randomly chosen delay period that was randomized for each visit and not known to the mouse. Incorrect port visits resulted in timeouts that were associated with a bright background across the entire arena. After each complete trial, which results in the reward state, mice are required to complete a separate task in which they need to ‘hunt’ for a series of 4 to 8 randomly placed blinking dots on the arena floor. Each dot disappears as soon as the mouse reaches it, resulting either in a new random annulus, or initialization of the next trial. The next trial begins with a new random rotation of the landmarks and rewarded port. (**c**) Training phases (see Methods). Mice are trained with a single landmark first, then 2 landmarks at unlimited view distance, and finally a limited view distance. (**d**) Top: Experimental setup for electrophysiology and real-time mouse position tracking. The arena was placed on top of a commercial flat-screen TV that was used to display visual landmarks. A motorized commutator^47^ was used to reduce tether-induced torque on the mouse, and a real-time optical tracking system was used to regulate the visibility of the landmarks and to identify when the mouse reached any of the blinking dots in the dot-hunting task. Bottom: view of the arena from the top, showing a subset of the reward ports as well as the tracking camera and the motorized commutator. (**e**) Example excerpt of behavioral data, with state transitions. Landmark visits (black arrowheads) are defined as the point when new landmarks become visible. (**f**) Top: Training curves for all 4 mice. The three major training phases are indicated with shading (corresponding to panel c). Red: Proportion of time that a landmark is visible (remains 1.0 (100%) until view distance is introduced). Blue: Maximum reward port hold time for each session, the actual hold times are drawn from a uniform distribution. Black: proportion of hits / false positives (corresponds to rewards / timeouts, or proportion correct), for the 1^st^ port visit in each trial. Values over 1/16 indicate that mice can distinguish the correct port amongst all ports. Values over 1 indicate that mice could reliably visit the correct port among the two ports indicated by locally ambiguous landmarks without excluding any other ports by trial and error (see main text and methods). Trials with 1^st^ landmark visit after <20 sec are included in analysis. Grey: Proportion of trials in which mice see both landmarks, and then turn around to go back to the 1^st^ landmark. If this proportion was 0, it would indicate that mice always visit the 2^nd^ port after seeing it, which would on average lead to chance-level behavioral performance. For each individual session, significance of correct choice for the 1^st^ port visit among the two indicated ports was tested with a binomial fit at the 95% level (two-sided, Clopper-Pearson exact method) and is indicated with a star. If the mouse also visited a large proportion of unmarked ports, this fraction can be significant despite the overall correct rate among all 16 ports being small. Bottom: latency to reward after encountering the 2^nd^ landmark in seconds (blue), proportion of visits to ‘a’ and ‘b’ as fraction of all port visits (orange) and proportion correct choice between ‘a’ or ‘b’ with binomial 95% CI (binomial as described before, green). See y-axis labels for unit definitions. (**g**) All paths taken by the mouse in one example session, split by LM0,1,2 state (green, glue, grey). (**h**) 6 example trials from the same session plotted from the start of the trial to the reward delivery, same color scheme as in g. 2 of the trials include time-outs (red). (**i**) Retrosplenial cortex is required for integrating egocentric sensory information and hypotheses about the animal’s allocentric location, but not for visually guided navigation. To causally test the role of RSC in relating spatial hypotheses to sensory data, we used a parametric allocentric/egocentric task using the same apparatus as in the main experiment and pharmacologically inactivated RSC. Schematic of task structure: Water restricted mice had to visit the port closest to a single visual landmark for a water reward. Visits to any other port resulted in a time-out, but allowed the mice to self-correct. As in the main experiment, the landmark and rewarded port were rotated randomly after each trial, forcing mice to use only the visual landmark. (**j**) To make the task reliant on allocentric hypotheses, we randomly varied the eccentricity of the landmark (center of the landmark to center of the arena, as fraction of the arena radius) at the beginning of each trial. Trials with low eccentricity (left) required the mouse to find the arena center (though path integration, requiring maintenance of a self-position hypothesis or memory in absence of persistence visual cues indicating the center of the arena) and then extrapolate a straight path through the landmark to the correct rewarded port. Alternatively, mice might triangulate which port is the closest to the landmark from the periphery. These strategies all require integration of self-location hypotheses with visual landmark information. Trials with high eccentricity (right) required merely walking to the port closest to the landmark. This design allowed us to test the role of RSC in the integration of location hypotheses with egocentric visual landmark information while simultaneously determining whether simpler visually-guided navigation was also affected. (**k**) RSC was either 1) transiently inactivated with Muscimol, 2) sham injected with cortex buffer, or 3) not injected (see methods). Each mouse was tested in both groups, with balanced ordering. (**l**) Task performance (mean and 95% confidence intervals for hit rate on 1^st^ port visits per trial, via binomial bootstrap). Mice always performed above full chance level (1/16^th^, assuming they cannot make use of the landmark). Performance was selectively reduced by RSC inactivation for low eccentricity conditions where integration of location hypotheses and visual landmarks was required. Performance in the visually guided condition was only minimally affected.

**Extended Data Fig. 2:**
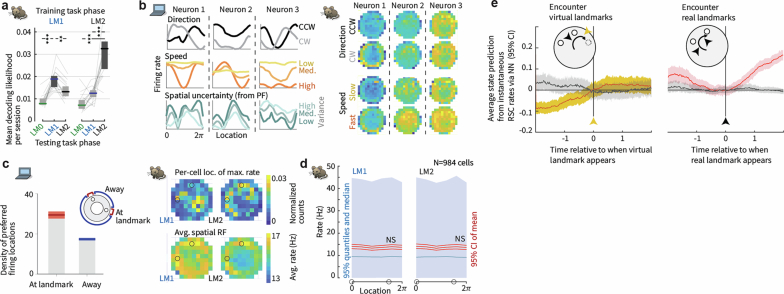
The spatial code in RSC changes with hypothesis states, ANN and RSC neurons employ conjunctive codes, and preferentially represent landmark / reward locations. (**a**) Location decoders (neural network, cross-validated per trial) do not generalize across landmark states, and LM1 carries less spatial information than LM2. Performance is measured by prediction likelihood in a 10×10 grid, means and shaded 95% CIs across sessions (N = 16 sessions). See Fig. 1f for test via spatial RF differences. (**b**) Left: Example ANN neuron tuning curves (from LM2) split by travel direction, speed, or location uncertainty (corresponding to LM0,1,2 states, derived from particle filter), showing conjunctive coding. Right: Three RSC example cells showing conjunctive coding of location vs. speed, and direction (Fig. 1d shows task phase vs. location). (**c**) Left: ANN neurons and, Right: RSC cells (N = 984 neurons) weakly preferentially fire at landmark locations. Top: distribution of locations where RSC cells fire most. Bottom: total average rates, split by LM1 and LM2. (**d**) Distribution of firing rates by angular position in the arena, same data as panel c. Blue: quantile of firing rates across population. Red: 95% CI of the mean across the population via bootstrap. Despite a small preference for the landmark locations, this effect is small compared to the overall variability on firing rates, and there is no systematic preference for cells to fire in proximity of one vs. the other landmark, even in the LM2 condition. (**e**) Information gain, which we study by analyzing landmark encounters, as transitions between LM_0,1,2_ states throughout the manuscript, can also occur when mice fail to encounter a landmark where one would be expected given some hypothesis (see Fig. 2e for an ANN example). These cases can also be decoded from neural activity, but cannot be directly compared to landmark encounters, as they don’t offer the matched sensory input (that is no visual input vs. appearance of salient landmark) that we employ in Fig. 4 (mouse encounters 2^nd^ landmark, but it is either ‘a’ or ‘b’). These ‘virtual’ landmark encounters were decoded with a cross-validated NN on a trial level and compared to real landmark encounters. The same decoder was then cross tested on the reverse condition (grey plot) to show that the neural code for encounters and non-encounters is different, as is expected from the different sensory inputs. Analysis as in Extended Data Fig. 9a, but plots are aligned to the value at the 0-second point, see Methods for details.

We trained freely moving mice to distinguish between two perceptually identical landmarks, formed by identical dots on a computer-display arena floor, by sequentially visiting them and reasoning about their relative locations. The landmarks were separated by <180 degrees in an otherwise featureless circular arena (50-cm diameter), to create a clockwise (CW) (‘a’) and a counterclockwise (CCW) (‘b’) landmark. Across trials, the relative angle between landmarks was fixed and the same relative port was always the rewarded one; within trials, the locations of landmarks was fixed. The mouse’s task was to find and nose-poke at the CCW ‘b’ landmark for water reward (‘b’ was near one of 16 identical reward ports spaced uniformly around the arena; other ports caused a time out). At most, one landmark was visible at a time (enforced by tracking mouse position and modulating landmark visibility based on relative distance (Extended Data Fig. 1; Methods). Each trial began with the mouse in the center of the arena in the dark (‘LM0’ phase; Fig. 1b), without knowledge of its initial pose. In the interval after first encountering a landmark (‘LM1’ phase), an ideal agent’s location uncertainty is reduced to two possibilities, but there is no way to disambiguate whether it saw ‘a’ or ‘b.’ After seeing the second landmark, an ideal agent could infer landmark identity (‘a’ or ‘b’; this is the ‘LM2’ phase; Fig. 1b) by estimating the distance and direction traveled since the first landmark and comparing those with the learned relative layout of the two landmarks; thus, an ideal agent can use sequential spatial reasoning to localize itself unambiguously. For most analyses, we ignored cases where mice might have gained information from not encountering a landmark, for example, as the artificial neural network (ANN) does in Fig. 2e (and Extended Data Fig. 2e). To randomize the absolute angle of the arena at the start of each new trial (and thus avoid use of any olfactory or other allocentric cues), mice had to complete a separate instructed visually guided dot-hunting task, after which the landmarks and rewarded port were rotated randomly together (Extended Data Fig. 1b).

Mice learned the task (*P* < 0.0001 on all mice, Binomial test versus random guessing; Fig. 1c), showing that they learn to form hypotheses about their position during the LM1 phase, retain and update these hypotheses with self-motion information until they encounter the second (perceptually identical) landmark, and use them to disambiguate location and determine the rewarded port. We hypothesized that RSC, which integrates self-motion^5^, position^6–8^, reward value^9^ and sensory^10^ inputs, could perform this computation. RSC is causally required to process landmark information^11^, and we verified that RSC is required for integrating spatial hypotheses with visual information but not for direct visual search with no memory component (Extended Data Fig. 1i–l).

### Spatial hypotheses are encoded conjunctively with other navigation variables in RSC

**Extended Data Fig. 3:**
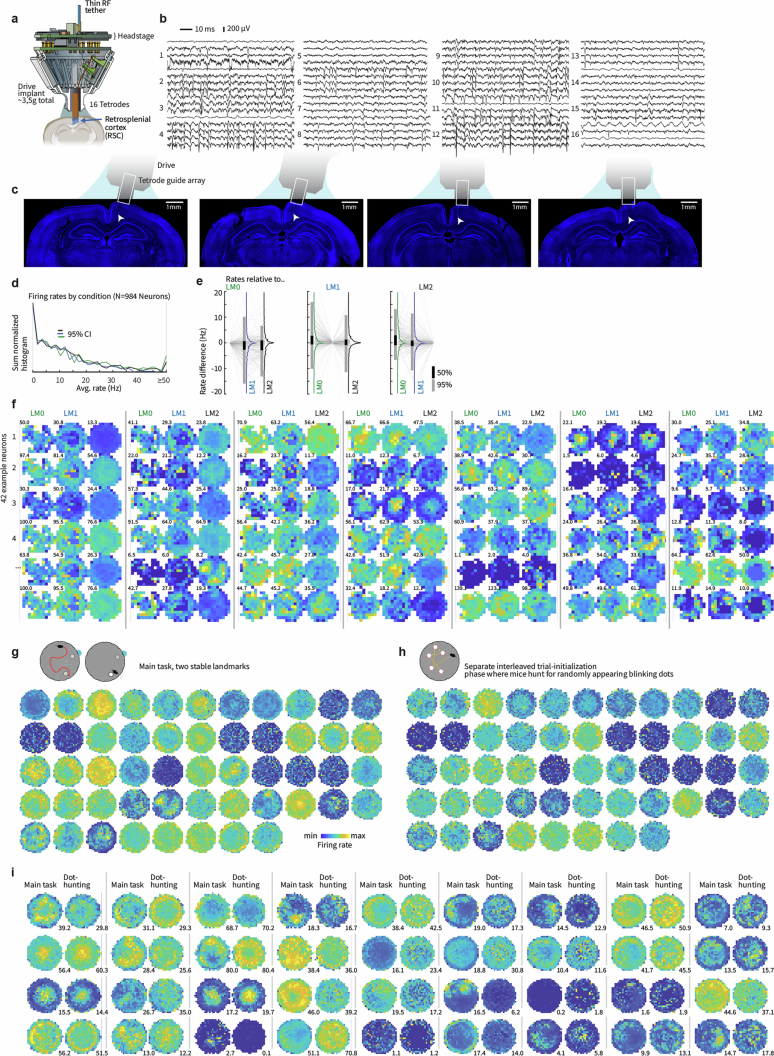
Extracellular recording in mouse retrosplenial cortex. (**a**) Tetrode drive^12^ implants targeting mouse retrosplenial cortex (RSC). See Methods for details. (**b**) Example band-passed (100Hz-5kHz) raw voltage traces from 16 tetrodes. (**c**) Verification of drive implant locations in RSC via histology in all 4 mice. White arrowheads indicate electrolytic lesion sites. (**d**) Histograms of mean firing rates of all 984 neurons across LM0 (green), LM1 (blue), and LM2 (black) conditions. Neurons are treated as independent samples. Overall rates did not shift significantly across these states. (**e**) Relative per-neuron changes in firing rates across conditions. Despite the lack of a population-wide shift in average rates, the firing rates of individual cells varied significantly across conditions with heterogeneous patterns of rates. Each grouping shows rates per cell, relative to the rate in LM0 (left) LM1 (middle), and LM2 (right) as individual rates (grey lines and histograms). Bar graphs show the 50% and 95% quantiles. (**f**) Spatial firing profiles of 42 example neurons split by hypothesis state. Number insets denote Max. firing rate in Hz per condition. For clarity, missing data is that was not due to exclusion via landmark visibility in LM1 is plotted as the darkest color in each plot. (**g**) Spatial firing rate profiles for all neurons from one example session (52 total), from the main task phase. Profiles were computed in 25×25 bins, and individually normalized to their 99^th^ percentile. (**h**) same as panel g, but from the separate trial initialization task (‘dot-hunting’) in which mice had to hunt for a series of blinking dots that appeared in random positions. (**i**) 36 example neurons from multiple sessions and animals, chosen to represent the broad range of tuning profiles. For each neuron, the main task tuning and the ‘dot-hunting’ are plotted together on the same brightness scale, normalized to their total maximal rate. In the dot-hunting task there is no conserved radial tuning due to the absence of consistent landmarks, however some cells retain angular spatial tuning due to olfactory cues in the arena. Tuning to eccentricity (distance to arena wall or center) is maintained across task phases in many neurons. Small numbers indicate maximum firing rates in Hz for each plot (color scale is same across the pairs).

We recorded 50–90 simultaneous neurons in layer 5 of RSC in four mice during navigational task performance using tetrode array drives^12^ and behavioral tracking (Fig. 1a and Extended Data Figs. 1 and 3; Methods). RSC neurons encoded information about both the mouse’s location (Fig. 1d) and about the task phase, corresponding to possible location hypotheses (Fig. 1d,e). This hypothesis encoding was not restricted to a separate population: most cells encoded both hypothesis state as well as the animal’s location (Fig. 1g).

**Extended Data Fig. 4:**
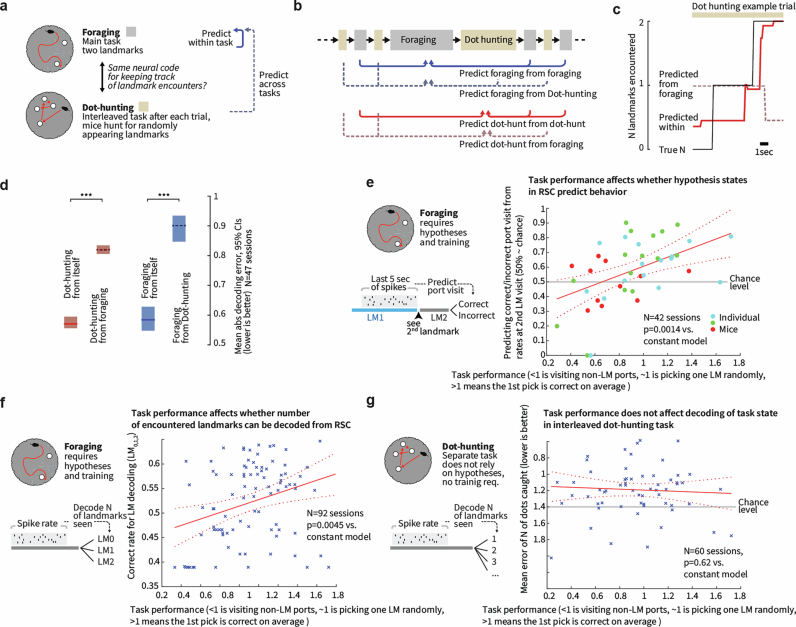
Hypothesis encoding in RSC is task-specific and is a function of task learning. (**a**) Foraging and dot-hunting tasks are interleaved, allowing comparisons of how the same neural population represents hypotheses. (**b**) We predict the number of encountered landmarks either within condition (for example foraging from foraging, each time using one trial as test, fitting to all others), or across. Only the first 2 landmarks were predicted to allow use of the same classifier across both despite the higher number of landmarks in the dot-hunting task. Train and test sets were split by trial. Decoding was done with a regression tree on low-pass filtered firing rates. Performance was quantified as mean error on the N of landmarks. (**c**) Example dot-hunting trial, the performance from using the foraging predictor is lower. (**d**) Summary stats from all sessions, means and bootstrapped CIs. The prediction is significantly better when using training data from the same category than when using the neural code from the other; for example dot-hunting to predict the foraging (P= ~0 / ~ 0 within vs. across categories for predicting dot-hunting and foraging landmark state), showing that hypothesis coding is task-specific. (**e**) To test whether hypothesis encoding is a specific function of task learning or a general feature of RSC, we examined whether coding persisted in case when mice performed the task but were not yet performing well. We first examined the ability to predict correct vs. incorrect port choice (same as in Fig. 4) as a function of per-session task performance. We analyzed data from sessions from the entire training period where the 2 landmarks were used, with at least 5 correct and 5 incorrect choices (N = 42 sessions), due to the closely spaced recordings, neurons might be re-recorded across sessions. On average we analyzed ~15-30 port visits per session (number of trials was unaffected by behavioral performance: CI of slope = [-7.7, 2.7], p = 0.33). Predictions were made as before with a test/train split on balanced hit/miss data with a regression tree. Prediction performance was at chance level ( ~ 47%, P = 0.81 vs. chance) for low performance sessions (total correct choice ratio of 0.8 or lower), and the same as in our initial analysis (Fig. 4) for sessions with high mouse performance ( ~ 66%, P = 0.00096 vs. chance). Overall, prediction performance was significantly correlated with task performance (P = 0.0014 vs. constant model). Individual mice are indicated with colored markers. (**f**) We also analyzed the more general decoding of landmark encounter count (same as Fig. 1) in all of the 92 sessions with 2 landmarks, and also found a significant correlation (p = 0.0045 vs. constant model), showing that hypothesis encoding throughout the task is driven by task learning. (**g**) As a control experiment, we tested whether decoding the number of landmarks encountered in the interleaved dot-hunting task might also be affected by task performance, if for instance the neural encoding and performance was a function of general spatial learning, habituation to the arena, motivation, etc., and we found this correlation to be flat (P = .6, CI for slope = [-0.17, 0.29]). We conclude that the encoding of hypothesis state is task-specific and a function of the mouse performing the task.

This encoding was distinct from the encoding of landmark encounters in the interleaved dot-hunting task and was correlated per session with behavioral performance (Extended Data Fig. 4). The encoding of mouse location changed significantly across task phases (Fig. 1d,f), similar to the conjunctive coding for other spatial and task variables in RSC^6^. This mixed co-encoding of hypothesis, location and other variables suggests that RSC can transform new ambiguous sensory information into unambiguous spatial information through the maintenance and task-specific use of internally generated spatial hypotheses.

### Hypothesis-dependent spatial computation using recurrent dynamics

**Extended Data Fig. 5:**
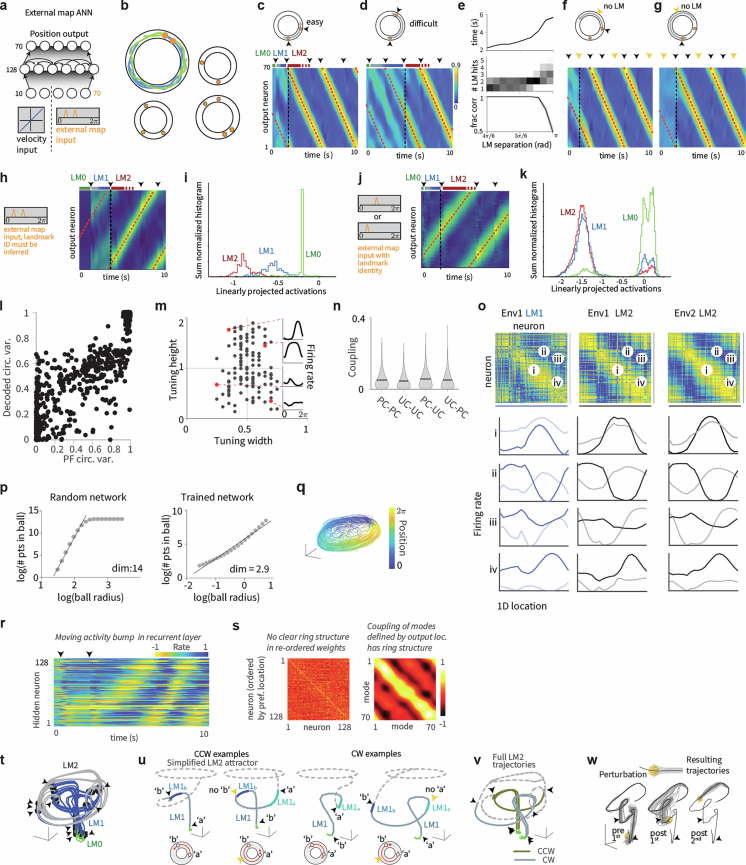
Architecture, trajectories, and population statistics for ANN with external map input. (**a**) Structure of the recurrent network. Input neurons encoded noisy velocity input with linear tuning curves (similar to speed cells in the entorhinal cortex^60^), and landmark information. In the standard setup (referred to as “external map”), the landmark input signaled the global configuration of landmarks (map). If there are K landmarks (all assumed to be perceptually indistinguishable), then whenever the animal encounters a landmark, the input provides a simultaneous encoding of all K landmark locations using spatially tuned input cells. Thus, the input encodes the map of the environment but does not disambiguate locations within it. This input can be thought of as originating from a distinct brain area that identifies the current environment and provides the network with its map. (**b**) Trajectories varied randomly and continuously in speed and direction. There were 2-4 landmarks at random locations. (**c**) Activity of output neurons ordered by preferred location as a function of time in an easy trial with two nearby landmarks and a constant velocity trajectory. Black arrows: landmark encounters. Thick black dashed line: time of disambiguation of location estimate in output layer. Thin red dashed line: true location. The network’s decision on when to collapse its estimate is flexible, and dynamically adapts the decision time to task difficulty: When the task is harder because of the configuration of landmarks (the task becomes harder as the two landmarks approach a 180 degree separation because of velocity noise and the resulting imprecision in estimating distances; the task is impossible at 180 degree because of symmetry), the network keeps alive multiple hypotheses about its states across more landmark encounters until it is able to reach an accurate decision. Panels c,d,f,g show example trials from experiment configuration 4 (See Methods) with different values of landmark separation parametrized by α. (**d**) Same as c, but in a difficult trial with two landmarks almost opposite of each other. (**e**) Top: The ANN took longer to disambiguate its location in harder task configurations: average time until disambiguation as a function of landmark separation (Standard error bars are narrower than line width). Middle: Distribution of the number of landmark encounters until the network disambiguates location, as a function of landmark separation. Bottom: Fraction of trials in which the network location estimate is closer to the correct than the alternative landmark location at the last landmark encounter, as a function of landmark separation. Data from 10000 trials in experiment configuration 4, 1000 for each of the 10 equally spaced values of α. The performance of the ANN (Fig. 2 main text) can be compared to the much poorer performance achieved by a strategy of path integration to update a single location estimate with landmark-based resets (to the coordinates of the landmark that is nearest the current path-integrated estimate), Fig. 2b (black versus gray). The latter strategy is equivalent to existing continuous attractor integration models^13,14^ combined with a landmark- or border based resetting mechanism^16,56,57,61^, which to our knowledge is as far as models of brain localization circuits have gone in combining internal velocity-based estimates with external spatial cues. The present network goes beyond a simple resetting strategy, matching the performance of a sequential probabilistic estimator – the particle filter (PF) – which updates samples from a multi-peaked probability distribution over possible locations over time and is asymptotically Bayes-optimal (M = 1000 particles versus N = 128 neurons in network; Fig. 2b, lavender (PF) and green (enhanced PF)). Notably, the network matches PF performance without using stochastic or sampling-based representations, which have been proposed as possible neural mechanisms for probabilistic computation^39,62^. (**f**) Similar to c, but in a trial where the network disambiguates its location before the second landmark encounter. Yellow arrows mark times of landmark interactions if the alternative location hypothesis had been correct. Disambiguation occurs shortly after the absence of a landmark encounter at the first yellow arrow. (**g**) Similar to f, but in a trial where disambiguation occurs at the first landmark location, since no landmark has been encountered at the time denoted by the first gray arrow. (**h**) In the regular task where landmark identity must be inferred by the ANN, discrete hypothesis states (denoted LM0,1,2 throughout) emerge during the LM1 state. (**j**) If the ANN is instead given the landmark identity via separate input channels, it immediately identifies the correct location after the 1^st^ landmark encounter and learns to acts as a simple path integration attractor without hypothesis states. Plots show ANN output as in c,d,g,f. (**i,k**) To quantify the separation of hypothesis states in the ANNs hidden states even in cases where such separation might not be evident in a PCA projection, we linearly projected hidden state activations onto the axis that separates the hypothesis states. The regular ANN shows a clear LM1 vs LM2 separation, but the ANN trained with landmark identity does not distinguish between these. (**l**) Population statistics for ANN with external map input. Scatter plot of enhanced particle filter (ePF) circular variance vs. estimate decoded from hidden layer of the network. 4000 trials from experiment configuration 1 were used to train a linear decoder on the posterior circular variance of the ePF from the activity of the hidden units and performance was evaluated on 1000 test trials. (**m**) Scatter plot of widths and heights of ANN tuning curves after the 2^nd^ landmark encounter. Insets: example tuning curves corresponding to red dots. Unlike hand-designed continuous attractor networks, where neurons typically display homogeneous tuning across cells^13,63,64^, our model reproduces the heterogeneity observed in hippocampus and associated cortical areas. Tuning curves are from LM2 using 1000 trials from experiment configuration 2 using 20 location bins. Tuning height specifies the difference between the tuning curve maximum and minimum, and tuning width denotes the fraction of the tuning curve above the mean of maximum and minimum. (**n**) The distribution of recurrent weights shows that groups of neurons with strong or weak location tuning or selectivity have similar patterns and strengths of connectivity within and between groups: distribution of absolute connection strength between and across location-sensitive “place cells” (PCs) and location-insensitive “unselective cells” (UCs) in the ANN. The black line denotes the mean; s.e.m. is smaller than the linewidth. The result is consistent with data suggesting that place cells and non-place cells do not form distinct sub-networks, but are part of a system that collectively encodes more than just place information^65^. Location tuning curves were determined after the second landmark encounter using 5000 trials from distribution 1 and using 20 location bins. The resulting tuning curves were shifted to have minimum value 0 and normalized to sum to one. The location entropy of each neuron was defined to be the entropy of the normalized location tuning curve. Neurons were split in two equal sets according to their location entropy, where neurons with low entropy were defined as “place cells” (PCs) and neurons with high entropy were defined as “non-place cells” (UCs). Between and across PCs and UCs absolute connection strength was calculated as the absolute value of the recurrent weight between non-identical pairs. (**o**) Pairwise correlation structure^30^ is maintained across LM[1,2] states and environments. Corresponds to Fig. 3a. Top: Correlations in spatial tuning between pairs of cells in one environment after the 1^st^ landmark encounter / LM1 (left), after the 2^nd^ encounter / LM2, and in a separate environment in LM2 (right). The neurons are ordered according to their preferred locations in environment 1. Bottom: Example tuning curve pairs (normalized amplitude) corresponding to the indicated locations i-iv. Data from experiment configuration 1. (**p**) State-space activity of ANN is approximately 3-dimensional. Even when summed across all environments and random trajectories, the states still occupy a very low-dimensional subspace of the full state space, quantified by the correlation dimension as d ≈ 3 (left, see Methods). This measure typically overestimates manifold dimension^66^, and serves as an upper bound on the true manifold dimension. As a control, the method yields a much larger dimension (d = 14) on the same network architecture with large random recurrent weights (right); thus, the low-dimensional dynamics are an emergent property of the network when it is trained on the navigation task. Data from 5000 trial, recurrent weights were sampled i.i.d. from a uniform distribution *W*_*h,ij*_ ~ *U*([ − 1, 1]), then fixed across trials. The initial hidden state across trials was sampled from h_*t=0,i*_ ~ *U*([ − 1, 1]). Data from 5000 trials from experiment configuration 1. (**q**) In the LM2 state, position on the rate-space attractor corresponds to location in the maze. State-space trajectories after second landmark encounter for random trajectories. Color corresponds to true location (plot shows 100 trials). (**r**) ANN with external map input implements a circular attractor structure: Hidden layer activity arranged by preferred location in an example trial shows a bump of activity that moves coherently. Black arrows: first two landmark encounters. Preferred location was determined after the second landmark encounter using 5000 trials from experiment configuration 1. (**s**) Left: Recurrent weight matrix arranged by preferred location of neurons (determined after the second landmark encounter using 5000 trials from experiment configuration 1) indicates no apparent ring structure, despite apparent bump of activity that moves with velocity inputs (panel a). Right: However, recurrent coupling of modes defined by output weights (defined by ${W}_{{out}}{W}_{{rec}}{W}_{{out}}^{T}$, where ${W}_{{rec}}$ are the recurrent weights and ${W}_{{out}}$ are the output weights) has a clear band structure. Connections between appropriate neural mixtures in the hidden layer – defined by the output projection of the neurons – therefore exhibit a circulant structure, but the actual recurrent weights do not, even after sorting neurons according to their preferred locations. The ANN thus implements a generalization of hand-wired attractor networks, in which the integration of velocity inputs by the recurrent weights occurs in a basis shuffled by an arbitrary linear transformation. Given these results, one cannot expect a connectomic reconstruction of a recurrent circuit to display an ordered matrix structure even when the dynamics are low-dimensional, without considering the output projection. Because trials in the mouse experiments typically ended almost immediately when the mouse had seen both landmarks (See Extended Data Fig. 1f for a quantification), we did not quantify the topology of the neural dynamics in RSC. (**t**) Low-dimensional state-space dynamics in the ANN with external map input suggests novel form of probabilistic encoding. Visualization of the full state-space dynamics of the hidden layer population, projected onto the three largest principal components, for constant-velocity trajectories. ANN hidden layer activity was low-dimensional: Fig. 3a shows data on low-dimensional dynamics, evident in maintained pairwise correlations, and Fig. 3d and panel p show correlation dimension. Trajectories are shown from the beginning of the trials; arrows indicate landmark encounter locations, black squares: first landmark encounter; black circles: second landmark encounter; line colors denote trajectory stage: LM0 (green), LM1 (blue), andLM2 (grey). Data in a-c is from 1000 trials from experiment configuration 3 (see Methods); sensory noise was set to zero. Trajectory starting points were selected to be a fixed distance before the first landmark. The intermediate ring (LM1) corresponds to times at which the output neurons represent multiple hypotheses, whereas the final location-coding ring (LM2), well-separated from the multiple hypothesis coding ring, corresponds to the period during which the output estimate has collapsed to a single hypothesis. In other words, the network internally encodes single-location hypothesis states separably from multi-location hypothesis states, as we find in RSC (Fig. 1), and transitions smoothly between them, a novel form of encoding of probability distributions that appears distinct from previously suggested forms of probabilistic representation^39,62^. (**u**) ANN trial trajectory examples, (corresponding to Fig. 2e). Divergence of trajectories for two paths that are idiothetically identical until after the second landmark encounter. ‘a’ and ‘b’ denote identities of locally ambiguous identical landmarks. Disambiguation occurs at the second landmark encounter, or by encountering locations where a landmark would be expected in the opposite identity assignments. See insets for geometry of trajectories and landmark locations. LM2 state has been simplified in these plots. (**v**) All four trajectories from panel b plotted simultaneously, and with full corresponding LM2 state. (**w**) The low-dimensional state-space manifold is stable, attracting perturbed states back to it, which suggests that the network dynamics follow a low-dimensional continuous attractor and the network’s computations are robust to most types of noise. Relaxations in state space after perturbations before the first (left), between first and second (middle), and after the second (right) landmark encounter. For the base trial, a trial with two landmarks and random trajectory was chosen. The first and second landmark encounter in this base trial is at time t = 2 s and t = 4.6 s respectively. At time t = 1 s (left), t = 4 s (middle), and t = 7 s (right) a multiplicative perturbation of size 50% was introduced at the hidden layer. See Extended Data Fig. 10l for same result on internal map ANNs.

To test whether recurrent neural networks can solve sequential spatial reasoning tasks that require hypothesis formation, and to provide insight into how this might be achieved in the brain, we trained a recurrent ANN on a simplified one-dimensional (1D) version of the task, since the relevant position variable for the landmarks was their angular position (inputs were random noisy velocity trajectories and landmark positions, but not their identity; Fig. 2b). The ANN performed as well as a near Bayes-optimal particle filter (Fig. 2b), outperforming path integration with correction (corresponding to continuous path integration^13,14^ with boundary/landmark resetting^15,16^) and represented multimodal hypotheses, transitioning from a no-information state (in LM0) to a bimodal two-hypothesis coding state (LM1) and finally to a full information, one-hypothesis coding state (LM2) (Fig. 2c,d and Extended Data Fig. 5). Bimodal hypothesis states did not emerge when the ANN was given the landmark identity (Extended Data Fig. 5h–k). Together, this shows that recurrent neural dynamics are sufficient to internally generate, retain and apply hypotheses to reason across time based on ambiguous sensory and motor information, with no external disambiguating inputs.

**Extended Data Fig. 6:**
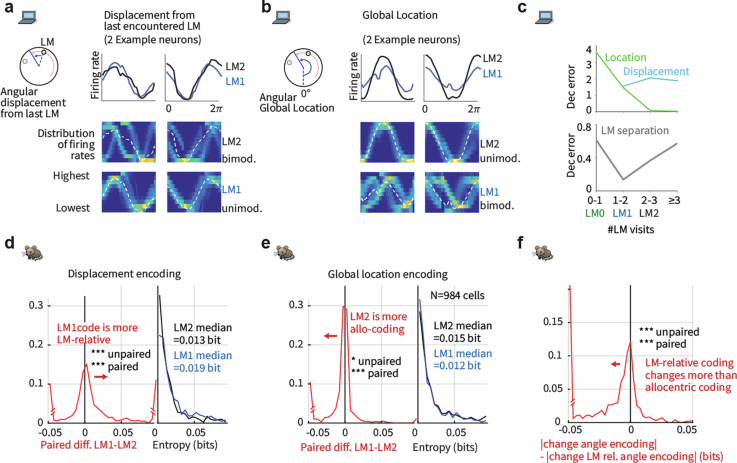
ANN and RSC coding transitions dynamically from an egocentric landmark-relative to an allocentric global reference frame based on phase in trial. (**a**) Top: Tuning curves (mean rate) for displacement from last encountered landmark for LM1 and LM2 states in ANN. Bottom: Same data, but distribution of firing rates. The network discovers that displacement from the last landmark encounter in the LM1 period is a key latent variable, and its encoding is an emergent property. Intriguingly, a similar displacement-to-location coding switch has been observed in mouse CA1^67^, suggesting that the empirically observed switch may be related to the brain performing spatial reasoning to disambiguate between multiple location hypotheses. (**b**) Same as panel a but for global location, ANN neurons became more tuned to global location rather than landmark-relative information after encountering the 2^nd^ landmark. (**c**) Decoding of location, displacement, and separation between landmarks from the ANN in a 2-landmark environment by a linear decoder that remains fixed across trials and environments. Top: Squared population decoding error of location (green) and displacement (blue), as a function of the number of encountered landmarks. As suggested by the well-tuned activity of ANN neurons, location can be linearly decoded in the LM2 state. Displacement can be best decoded in the LM1 state. Bottom: Square decoding error of distance between landmarks, as a function of the number of encountered landmarks. The representation is particularly accurate around the time just before and after the first landmark encounter, when location disambiguation takes place. Top: Performance was evaluated on 1000 trials from experiment configuration 2. For location, the decoder corresponded to the network location estimate. For displacement, the linear decoder was trained on 4000 separate trials. Bottom: experiment configuration 1 with 4000 trials to train the linear decoder and 1000 trials to evaluate it. Thus, the network’s encoding of these three critical variables is dynamic and tied to the different computational imperatives at each stage: displacement and landmark separation are not explicit inputs but the network estimates these and represents them in a decodable way at LM1, the critical time when this information is essential to the computation. After LM2, the network decodability of landmark separation drops, as it is no longer essential. (**d**) Neurons in RSC also became less well tuned to relative displacements from landmarks in LM2 relative to LM1: histogram across all RSC neurons of entropy of tuning curve for angular displacement from last seen landmark in RSC. Black: for LM2 state, Blue: for LM1 state. Red: histogram of pairwise differences. For this analysis, angular firing rate distributions were analyzed relative to either the global reference frame or the last seen landmark. (**e**) Same as d, but for global location. (**f**) The absolute change in landmark-relative displacement coding (d) is larger than that of the allocentric location tuning (e), suggesting that the latter is less affected by task state.

**Extended Data Fig. 7:**
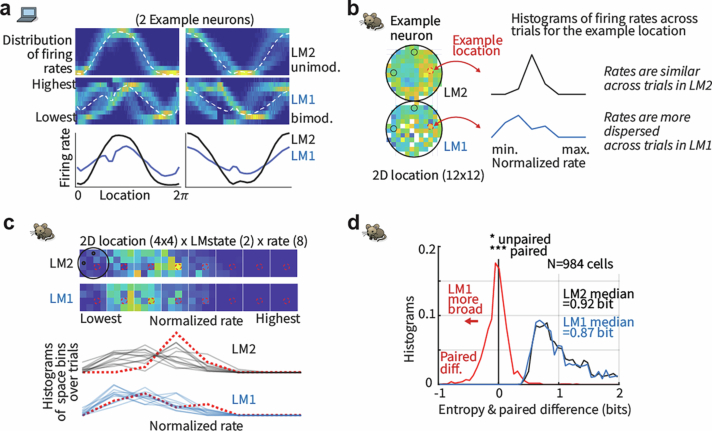
In addition to explicitly encoding number of visited landmarks, RSC and the ANN exhibit higher trial-to-trial variability in partial information states. (**a**) Bottom: Mean spatial activity profile of 2 example ANN neurons for LM1 and LM2. Average tuning is higher for the LM2 state. Top: same data as histograms, showing that the less well-tuned LM1 state corresponds to a bimodal rate distribution (rates are high in some trials, low in others) that transitions to a unimodal distribution once the 2^nd^ landmark has been identified in LM2. Data are from experiment configuration 2 (See Methods, section ‘Overview over experiment configurations used with ANNs’). Tuning curves were calculated using 20 bins of location/displacements and normalized individually for each neuron. The first time step in each trial and time steps with non-zero landmark input were excluded from the analysis. For histograms, each condition was binned in 100 column bins and neuron rates in 10 row bins. Histograms were normalized to equal sum per column. (**b**) Similarly, RSC rates are more dispersed per location in LM1. Schematic of analysis: firing rates were low pass filtered at 0.5 Hz, and for each location, the distribution of rates was computed in 8 bins, between the lowest and highest rate of that cell. (**c**) Example analysis for one cell. Top: Rate distribution resolved by 2D-location (4×4 bins) for example RSC neuron. Bottom: the resulting 16 histograms for LM1 and LM2 each, red dotted example histograms correspond to indicated example location (red dotted circles). (**d**) Summary statistics showing a more dispersed rate distribution per location in LM1. In sum, this analysis shows that in addition to the explicit encoding of uncertainty by a stable rate code (conjunctive with position and other variables), as shown in Fig. 1d,e,f and Extended Data Fig. 2a, where one would not expect a higher degree of trial-to-trial variability with higher uncertainty, there is still a degree of increased variability in states where the mouse might ‘take a guess’ that would differ between trials. This parallels a similar behavior in the ANN (panel a).

Both ANN and RSC neurons encoded several navigation variables conjunctively (Extended Data Fig. 2b) and transitioned from encoding egocentric landmark-relative position during LM1 to a more allocentric encoding during LM2 (Extended Data Fig. 6). Instantaneous position uncertainty (variance derived from particle filter) could be decoded from ANN activity (Extended Data Fig. 5l), analogous to RSC (Fig. 1e). ANN neurons preferentially represented landmark locations (Extended Data Fig. 2c; consistent with overrepresentation of reward sites in hippocampus^17,18^), but we did not observe this effect in RSC. Average spatial tuning curves of ANN neurons were shallower in the LM1 state relative to LM2, corresponding to trial-by-trial ‘disagreements’ between neurons, evident as bimodal rates per location. RSC rates similarly became less variable across trials per location in LM2 (Extended Data Fig. 7), indicating that, in addition to the explicit encoding of hypotheses/uncertainty (Fig. 1e,g), there is a higher degree of trial-to-trial variability in RSC as a function of spatial uncertainty.

**Fig. 3:**
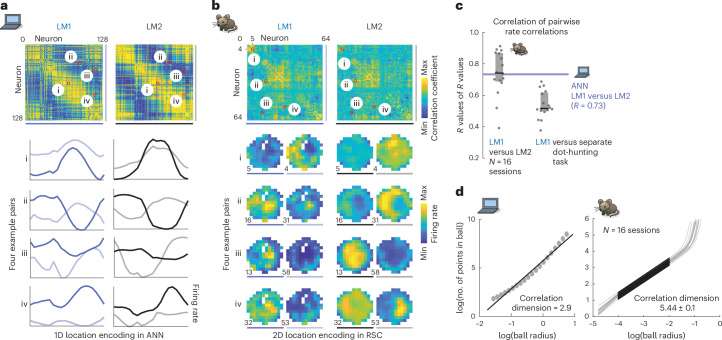
Stable low-dimensional dynamics for hypothesis-based stimulus disambiguation. **a**, Correlation structure in ANN activity is maintained across task phases, indicating maintained low-dimensional neural dynamics across different computational regimes. Top, pairwise ANN tuning correlations in LM1 and LM2 (same ordering, by preferred location). Bottom, tuning curve pairs (normalized amplitude). **b**, Same analysis as **a**, but for RSC in one session (*N* = 64 neurons, computed on entire spike trains, sorted via clustering in LM1). The reorganization of spatial coding as hypotheses are updated (Fig. 1d,f) is constrained by the stable pairwise structure of RSC activity. Neurons remain correlated (first and second pair) or anticorrelated (third and fourth pair) across LM1 and LM2. **c**, Summary statistics (session median and quartiles) for maintenance of correlations across task phases. This also extends to a separate visually guided dot-hunting task (Extended Data Fig. 8). **d**, Activity in both the ANN and RSC is locally low-dimensional, through correlation dimension (the number of points in a ball of some radius grows with radius to the power of N if data is locally N-dimensional) on 20 principal components. See Extended Data Fig. 8 for analysis by PCA.

The ANN computed, retained and used multimodal hypotheses to interpret otherwise ambiguous inputs: after encountering the first landmark, the travel direction and distance to the second is sufficient to identify it as ‘a’ or ‘b’ (Figs. 1b and 2a). There are four possible scenarios for the sequence of landmark encounters: ‘a’ then ‘b’, or ‘b’ then ‘a’, for CW or CCW travel directions, respectively. To understand the mechanism by which hypothesis encoding enabled disambiguation, we examined the moment when the second landmark becomes visible and can be identified (Fig. 2a). We designate LM1 states in which the following second landmark is ‘a’ as ‘LM1_a_’ and those that lead to ‘b’ as ‘LM1_b_.’ Despite trial-to-trial variance resulting from random exploration trajectories and initial poses, ANN hidden unit activity fell on a low-dimensional manifold (correlation dimension *d* ≈ 3; Fig. 3d) and could be well captured in a three-dimensional (3D) embedding using principal component analysis (PCA) (Fig. 2d). Activity states during the LM0,1,2 phases (green, blue and gray/red, respectively) were distinct, and transitions between phases (mediated by identical landmark encounters; black arrows) clustered into discrete locations. Examining representative trajectories (for the CCW case; Fig. 2e) reveals that LM1_a_ and LM1_b_ states are well-separated in activity space. If the second landmark appears at the shorter CCW displacement (corresponding to the ‘a’ to ‘b’ interval), the state jumps to the ‘b’ coding point on the LM2 attractor (Fig. 2e). On the other hand, the absence of a landmark at the shorter displacement causes the activity to traverse LM1_a_, until the second landmark causes a jump onto the ‘a’ coding location on the LM2 attractor. In both cases, an identical transient landmark input pushes the activity from distinct hypothesis-encoding regions of activity space onto different appropriate locations in the LM2 state, constituting successful localization.

We next consider the nature of the dynamics and representation that allows the circuit to encode the same angular position variables across LM1 and LM2 regimes while also encoding the different hypotheses required to disambiguate identical landmarks. Does the latter drive the network to functionally reorganize throughout the computation? Or, does the former, together with the need to maintain and use the internal hypotheses across time, require the network to exhibit stable low-dimensional recurrent attractor dynamics? To test this, we computed the pairwise correlations of the ANN activity states (Fig. 3a) and found them to be well conserved across LM1 and LM2 states. As these correlation matrices are the basis for projections into low-dimensional space, this shows that the same low-dimensional dynamics were maintained, despite spanning different computational and hypothesis-encoding regimes (metastable two-state encoding with path integration in LM1 versus stable single-state path integration unchanged by further landmark inputs in LM2; Extended Data Fig. 5). Low-dimensional pairwise structure was also conserved across different landmark configurations and varied ANN architectures, and the low-dimensionality of ANN states was robust to large perturbations (Extended Data Fig. 5w). In sum, these computations were determined by one stable set of underlying recurrent network dynamics, which, together with appropriate self-motion and landmark inputs, can maintain and update hypotheses to disambiguate identical landmarks over time, with no need for external inputs.

### RSC fulfills requirements for hypothesis-dependent spatial computation using recurrent dynamics

**Extended Data Fig. 8:**
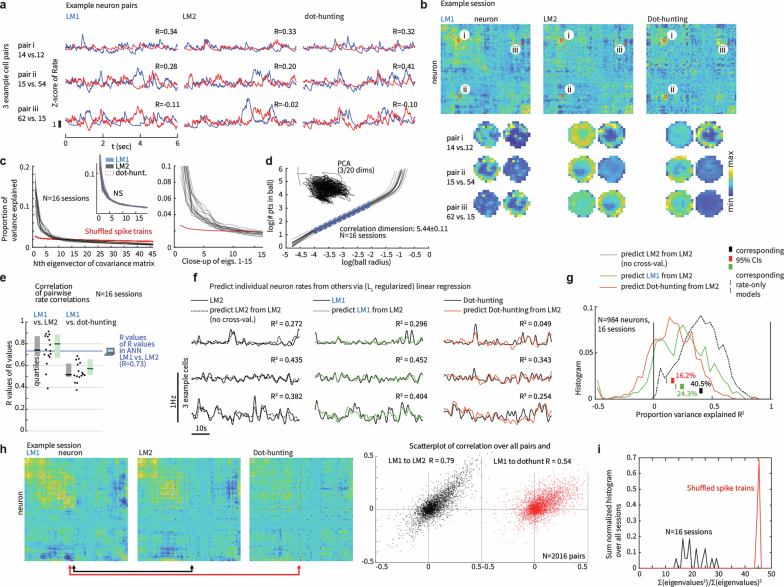
Pairwise rate correlation structure in RSC is maintained across LM1 and LM2 states. (**a**) Low-dimensional population structure can be probed by pairwise neural relationships^30^: correlations or offsets in spatial tuning between cell pairs should be preserved across environments if the dynamics across environments is low-dimensional. Example spike rates (6 sec window, low-passed at 1 Hz using a single-pole Butterworth filter) for 3 RSC neuron pairs from one example session. R values for each pair were computed across the LM1 and LM2 condition, as well as in the task-initialization phase where mice had to hunt blinking dots (Extended Data Figs. 1, 4). The latter provides a control condition where no landmark-based navigation was required and mice instead had to walk to randomly appearing targets. (**b**) Top: pairwise correlation matrices for LM1,2 and dot-hunting conditions. Example pairs are highlighted (i,ii,iii). Bottom: spatial firing rate profiles for example pairs. Same analysis as in Fig. 3a. (**c**) RSC activity is globally low-dimensional. Proportion of variance of low-pass filtered (0.5 Hz) firing rates explained by first 45 principal components from the LM1 states. Proportion of variance explained (black, 16 sessions) drops to below that of shuffled spike trains (red) after the 6-10^th^ principal component. The inset shows the analysis split by condition (same as in panels a and b), and 95% Cis for the spectra across sessions. The right panel shows zoomed in region of the same plot. We found no relationship between individual PCA components and task variables. (**d**) Correlation dimension in RSC is also low (same analysis as for the ANN in Extended Data Fig. 5p). This measure typically overestimates manifold dimension^66^, and thus serves as an upper bound on the true manifold dimension. (**e**) Grey/black: Summary statistics (median and quartiles) for correlation of correlations (panel b shows one example session, black dots indicate individual sessions, N = 16). Median of R value of R values for LM1 vs. LM2 = 0.74 (corresponding R in ANN = 0.73), for LM1 vs. dot-hunting = 0.51. Green: same analysis but spike rates were computed with a 5 Hz low-pass instead of the 1 Hz used throughout, no systematic changes were observed as function of low-pass settings. (**f**) Rates of individual RSC neurons can be predicted from other neurons with linear regression. In the LM2 to LM2 condition (black), the linear fit is computed for one held-out neuron’s rate from other concurrent rates, and the same regression weights are then used to predict rates during LM1 (green) and dot-hunting (red) time periods. True rates of predicted neurons are plotted as solid black lines. (**g**) Summary statistics for the linear regression. Histograms show the proportion of explained variance for all 984 neurons, split by condition. In the LM2 to LM2 condition, the fit is computed from other concurrent rates (40.5% variance explained, median across neurons). In the two other conditions, the regression weights are fit in LM2 and held fixed. The sequential, non-interleaved nature of this train/test split across task phases means that any consistent firing rate drifts across the conditions will lead to poor predictions, and consequently, a small number of neurons exhibit negative R^2^ values indicating a fit that can, for some cells, be worse than an average rate model (11.3% for LM1, 19.3% median across neurons for dot-hunting, small grey bars). However, 24.3% of variance (median across neurons) can be explained despite significant changes in spatial receptive fields (predict LM1 with LM2 weights) and even for a different task, with 16.2% when predicting dot-hunting activity from LM2 weights (red and green histogram and bars showing 95% CI of median). **(h)** Pairwise correlations between RSC neurons in another example session, same analysis as in panel b, and associated scatterplots. **(i)** Low-dimensional activity quantified via participation ratio (PR)^68^. This analysis does not account for noisy eigenvalue estimates from spiketrains, and consequently the shuffled spike trains where there are no prominent modes that correspond to stable sensory, motor, or latent states, yield values of PR = ~ 45.

We hypothesized that RSC and its reciprocally connected brain regions may, similarly to the ANN, use internal hypotheses to resolve landmark ambiguities using recurrent dynamics. Using the ANN as a template for a minimal dynamical system that can solve the task (Fig. 2), we asked whether neural activity in RSC is consistent with a system that could solve the task with the same mechanisms. To be described as a dynamical system, neural activity must first be sufficiently constrained by a stable set of dynamics, that is, the activity of neurons must be sufficiently influenced by that of other neurons, and these relationships must be maintained over time^1^. To test this property, we first computed pairwise rate correlations and found a preserved structure between LM1 and LM2, as in the ANN (median *R* (across sessions) of *R*s (across cells) = 0.74 in RSC, versus 0.73 in ANN; Fig. 3c). Firing rates could be predicted from rates of other neurons, using pairwise rate relationships across task phases; this maintained structure also extended to the visual dot-hunting behavior (Extended Data Fig. 8). Because pairwise correlations form the basis of dimensionality reduction, this shows that low-dimensional RSC activity is coordinated by the constraints of stable recurrent neural dynamics and not a feature of a specific behavioral task or behavior.

To employ neural firing rates as states of a dynamical system that act as memory and computational substrates in the same manner as in the ANN, they should also be low-dimensional. Consistent with the stable relationships between neurons, most RSC population activity was low-dimensional (around six significant principal components, and correlation dimension of around 5.4; Fig. 3d and Extended Data Fig. 8), similar to findings in hippocampus^19^. Together, we find that despite significant changes in neural encoding as different hypotheses are entertained across task phases (Fig. 1d–f and Extended Data Figs. 3f and 2a) and across different tasks (Extended Data Fig. 4a–d), the evolution of firing rates in RSC is constrained by stable dynamics that could implement qualitatively similar states as the ANN.

**Extended Data Fig. 9:**
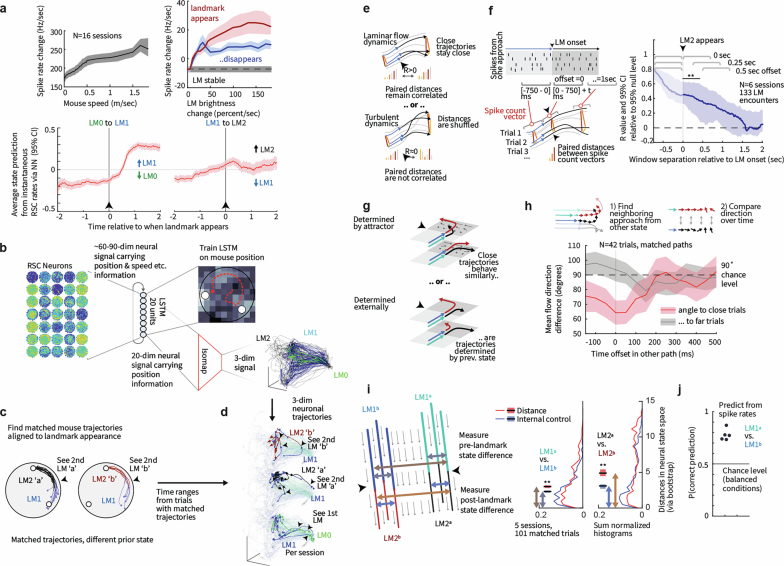
Low-dimensional spatial modes of mouse RSC activity. (**a**) Top left: the speed at which the neural activity evolves (avg. speed of largest 5 principal components, filtered at 1 Hz, CIs via bootstrap) correlates with running speed. Top right: When landmarks appear/disappear, they perturb neural activity (effect of mouse speed is regressed out). Bottom: Analysis of the time course of the prediction of LM0,1,2 state from RSC firing rates around the time when the landmarks appeared. Plots show 95% CIs for the mean of the state prediction, aligned to the mean, corresponding to a de-biased state estimation probability over time. Decoding was performed using the same method as in Fig. 1. (**b**) For some analyses of the low-dimensional dynamics in RSC (Fig. 4, this figure panel h), rate fluctuations related to non-spatial covariates such as speed, heading, etc. were removed: a single-layer LSTM with 20 hidden units was trained to predict the mouse position in a 10×10 grid from the RSC rates. The network learned 20 spatially relevant mixtures of input firing rates, with appropriate temporal smoothing to represent the mouse location. These activations were then embedded into 3-D space via isomap^53^. (**c**) To find trials across which mouse trajectories as they approached the 2^nd^ landmark were similar, mouse trajectories were clustered (see Methods) leading to a subset of trials with similar locomotion and visual inputs. (**d**) The activity of RSC, in the low-dimensional representation, and in raw spike counts was then analyzed further. The example plot shows low-dimensional neural trajectories from LM0,1,2 states during matched mouse trajectories. (**e**) Alternative hypotheses for smoothness / predictability of neural dynamics across trials (corresponding to Fig. 4c). Dynamics across trials could behave like a laminar flow, so that trials with similar neural state remain so (top), or they could shuffle, leading to a loss of the pairwise distance relationships across trials (bottom). (**f**) We measured this maintenance vs. loss of correlation in a sliding 750 ms window beginning at the 2^nd^ landmark onset, versus a window just before. CIs were computed across sessions (See Methods). (**g**) Hypotheses for whether stable neural dynamics (Fig. 3b,c, Extended Data Fig. 8) can determine how RSC activity encodes disambiguated landmark identity (‘a’ or ‘b’). Top: trials in which the correct identity is ‘a’ but that are neurally close to other trials where the answer is ‘b’ might get dragged along in the wrong direction at least transiently. This would indicate relevance of recurrent dynamics on this computation. Bottom: alternatively, neural activity could be determined by the correct answer, even in trials that (in neural rate space) are close to trials from the opposing class. (**h**) We tested this by finding the closest trial from the opposing class (for example the closest LM1_a_ for a LM1_b_ trial) in the 3-D embedded (via Isomap) RSC rate space. To evaluate co-evolution regardless of this selection confound, we then analyzed the direction of flow of the neural state over time (red). As a control, we also analyzed neurally far trials (grey). The flow direction of the neural activity was significantly aligned for ~100 ms. Median and CI via bootstrap. (**i**) Left: Schematic for the analysis of representation of LM1_a_ vs. LM1_b_ states. Trial-to-trial distances were compared within group vs. across group. Right: Both before and after the 2^nd^ landmark becomes visible, the classes are distinct in neural state space. (Same data as in Fig. 4b, 5 sessions, 101 matched trials). (**j**) Whether a trial comes from LM1_a_ or _b_ can also be decoded from low-pass filtered (2 Hz) firing rates before the 2^nd^ landmark onset (via regression tree, cross-validated across trials, balanced N across conditions, 5 sessions).

To compute with a dynamical system, states that act as memory need to affect how the system reacts to further input. The ANN solves the task using distinct hypothesis states that are updated with visual inputs and locomotion, by placing them in the state space so that visual input arriving at different hypothesis states within LM1 (LM1_a_ versus LM1_b_) pushes activity onto the correct states in LM2 (Fig. 2). We examined this process in RSC by first looking at the evolution of neural states during the spatial reasoning process. States evolved at speeds correlated with animal locomotion, consistent with the observation that hypotheses are updated by self-motion in between landmark encounters and were driven by landmark encounters consistent with findings in head-fixed tasks^11^ (Extended Data Fig. 9a). Neural states were also driven by failures to encounter landmarks at expected positions, which can also be informative (Fig. 2e, right), albeit with a different neural encoding than we observed for encountering the landmarks (Extended Data Fig. 2e).

**Fig. 4:**
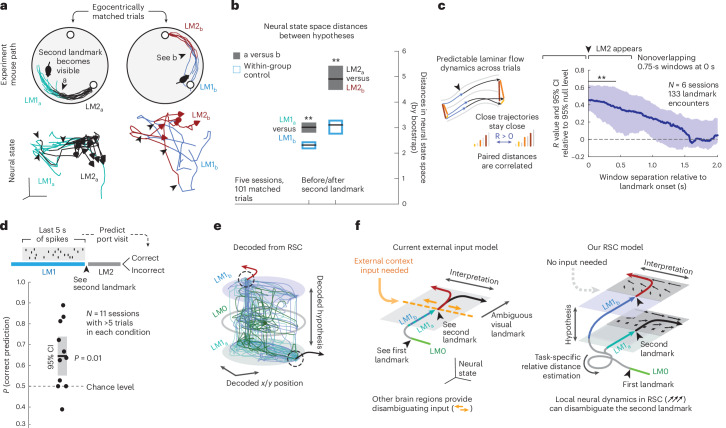
RSC exhibits stable attractor dynamics sufficient for computing hypothesis-dependent landmark identity. **a**, Top, to study hypothesis encoding and its impact without sensory or motor confounds, we used trials with matched egocentric paths just before and after the second landmark (‘a’ or ‘b’) encounter. One example session is shown. Bottom, 3D neural state space trajectories (isomap); RSC latent states do not correspond directly to those of the ANN. **b**, RSC encodes the difference between LM1_a_ and LM1_b_, and between subsequent LM2 states, as in the ANN (Fig. 2e and Extended Data Fig. 5). Blue, within-group and grey, across-group distances in neural state space. Horizontal lines, mean; boxes, 95% CIs (bootstrap). State can also be decoded from raw spike rates (Extended Data Fig. 9j). **c**, Neural dynamics in RSC are smooth across trials: pairwise distances between per trial spike counts in a 750 ms window before LM2 onset remain correlated with later windows; line, median; shading, CIs (bootstrap). **d**, RSC activity preceding the second landmark encounter predicts correct/incorrect port choice (horizontal line, mean; gray shaded box, 95% CI from bootstrap, cross-validated regression trees). **e**, Decoding of hypothesis states and position from RSC using ANNs to illustrate the evolution of neural activity in the task-relevant space (see **b**, **c** and **d** and Fig. 1e,f, Extended Data Fig. 9 statistics). **f**, Schematic of potential computational mechanisms. Left, if RSC encodes only current spatial and sensorimotor states and no hypotheses beyond landmark count (LM1_a_ or LM2_b_, derived from seeing the first landmark and self-motion integration that lead to identifying the second landmark as ‘a’ or ‘b’), an external disambiguating input is needed. Right, because task-specific hypotheses arising from the learned relative position of the landmarks are encoded (this figure), and activity follows stable attractor dynamics (Fig. 3), ambiguous visual inputs can drive the neural activity to different positions, disambiguating landmark identity in RSC analogously to the ANN.

We next tested whether sufficiently separated neural states, LM1_a_ and LM1_b_, together with stable low-dimensional attractor dynamics could resolve the identity of the second landmark. If so, this would suggest that, as in the ANN, the ensemble activity state in RSC can serve both as memory and affect future computations. We identified subsets of trials in which mouse motion around the LM1 to LM2 transition was matched closely and aligned them in time to the point when the second landmark became visible (Fig. 4a). In these trials, locomotion and visual inputs are matched, and only the preceding hypothesis state (LM1_a_ or _b_) differs. RSC firing rates differed between LM1_a_ and LM1_b_ states, as did subsequent rates in LM2 (comparing within- to across-group distances in neural state space across matched trials, and by decoding state from firing rates: Fig. 4b and Extended Data Fig. 9i,j).

To compute with the same mechanism as the ANN, neural states must be governed by stable dynamics consistently enough for current states to reliably influence future states, which requires that nearby states do not diffuse or mix too quickly^1^. We found that RSC firing rates were predictable across trials such that neighboring trials in activity space remained neighbors (Fig. 4c), which further confirms stable recurrent dynamics, that these states can be used as computational substrate, and indicates a topological organization of abstract task variables^19^. This indicates that stably maintained hypothesis-encoding differences in firing over LM1 could interact with ambiguous visual landmark inputs to push neural activity from distinct starting points in neural state space to points that correspond to correct landmark interpretations, as in the ANN.

The ANN achieved high correct rates, but mice make mistakes. If the dynamical systems interpretation holds, such mistakes would be explainable by LM1_a_ or _b_ states that are not in the right location, and lead to the wrong LM2 interpretation. Indeed, we observed that neural trajectories from LM1_a_ that were close in activity space to LM1_b_ were dragged along LM1_b_ trajectories and vice-versa (they had similar movement directions; Extended Data Fig. 9g,h), suggesting that behavioral landmark identification outcomes might be affected by how hypotheses were encoded in RSC during LM1. We tested this hypothesis and found that RSC activity in LM1 (last 5 s preceding the transition to LM2) was predictive of the animal’s behavioral choice of the correct versus incorrect port (Fig. 4d). Notably, this behaviorally predictive hypothesis encoding was absent during training in sessions with low task performance (Extended Data Fig. 4), indicating that the dynamical structures and hypothesis states observed in RSC were task-specific and acquired during learning.

Our unrestrained nonstereotyped behavior is not amenable to direct comparison of activity trajectories between ANNs and the brain as others have done in highly stereotyped trials of macaque behavior^1^. Instead, we found that the dynamics of firing rates in mouse RSC are consistent with, and sufficient for, implementing hypothesis-based disambiguation of identical landmarks using a similar computational mechanism as observed in the ANN.

## Discussion

**Extended Data Fig. 10:**
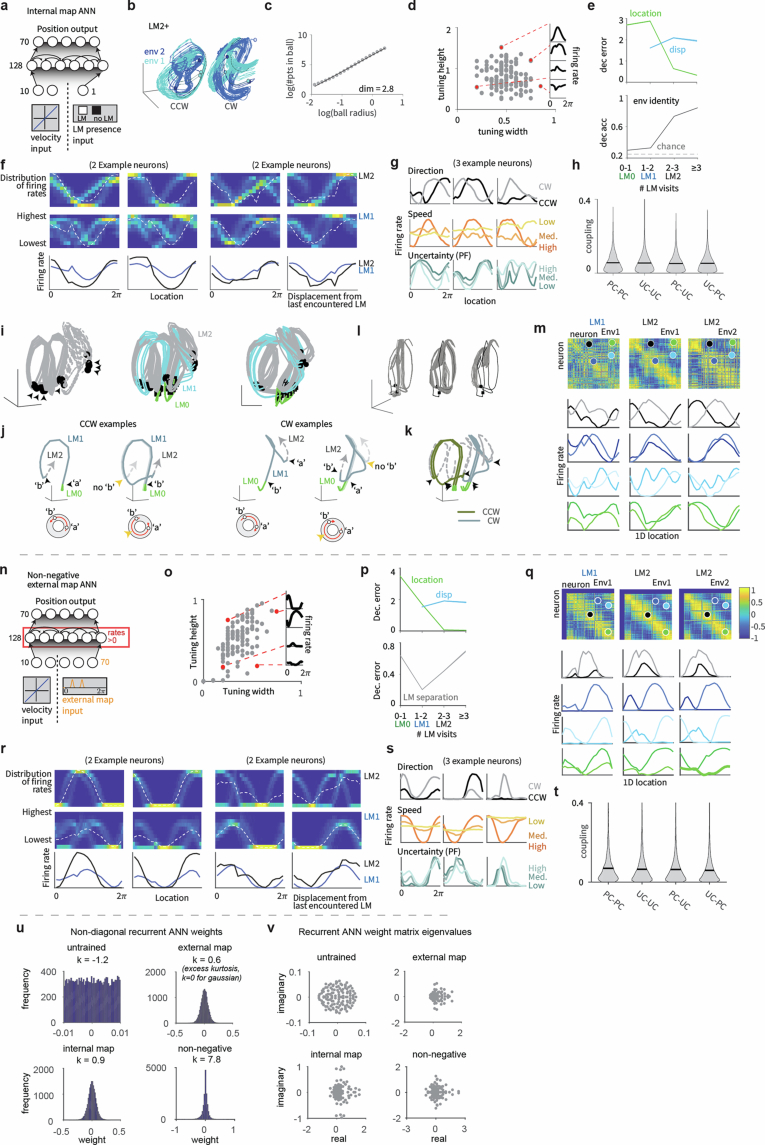
ANN with binary landmark presence input, and ANN with non-negative rates, recapitulates all main findings from the external map ANN. (**a**) ANN with binary landmark presence input. Here, the ANN must simultaneously infer the landmark locations and the location of the animal, in contrast to the previous “external map” configuration. These determinations are inter-related, thus the much higher difficulty of the task. Structure of the recurrent network. Input neurons encoded noisy velocity (10 neurons) and landmark information (1 neuron). In the internal map setup, the input signaled whether a landmark was present at the current position or not. (**b**) State space trajectories in the internal map network after the second landmark encounter in two different environments. The dark green / dark blue parts of the trajectories correspond to the sections before the third landmark encounter. Left: Predominantly counterclockwise trajectories, right: Predominantly clockwise trajectories. Landmarks and trajectories were sampled with the same parameters as experiment configuration 1, but the duration of test trials was extended from 10 s (100 timesteps) to 50 s (500 timesteps). Only trials with low error after the second landmark encounter are shown, defined as maximum network localization error smaller than 0.5 rad, measured in a time window between 5 timesteps after the second landmark encounter until the end of the trial. Only the state-space trajectory after the second landmark encounter is displayed. (**c**) State space dimension is approximately 3, same analysis as in Extended Data Fig. 5p. (**d**) Example tuning curves, same analysis as in Extended Data Fig. 5m. (**e**) Linear decoding of position, displacement from last landmark and landmark separation from ANN activity, same analysis as in Extended Data Fig. 6c. A multinomial regression decoder was trained on 4000 trials from experiment configuration 1 (the training distribution of the internal map task) to predict from hidden layer activities which of the four possible environments was present. Performance was evaluated on separate 1000 test trials sampled from the training distribution. (**f**) Example neurons showing transition from egocentric landmark-relative displacement coding to allocentric location encoding, same analysis as in Extended Data Fig. 6a,b. (**g**) Example neurons showing conjunctive encoding, same analysis as in Extended Data Fig. 2b. Location tuning curves were determined after the second landmark encounter using 1000 trials from experiment configuration 2 using 20 location bins. Velocity and uncertainty from the posterior circular variance of the enhanced particle filter were binned in three equal bins. (**h**) Distribution of absolute connection strength between and across location-sensitive “place cells” (PCs) and location-insensitive “unselective cells” (UCs), same analysis as in Extended Data Fig. 5n. (**i)** Hidden unit activations, corresponding to Fig. 2d. (**j**) Trajectories from example trials, as in Fig. 2e. (**k**) Same trajectories as in i&j but with full LM2 state. (**l**) ANN is robust to perturbations, same as in Extended Data Fig. 5w. (**m**) ANN maintains pairwise correlation structure across states and environments, same as in Fig. 3a and Extended Data Fig. 5o. (**n**) ANN with non-negative rates recapitulates the main findings from the conventional ANNs. Training an ANN in the external map condition but with non-negative activity replicated all key results from the other NN types: we observed similar results with respect to location and displacement tuning (r), the transition in linear decodability of displacement to location from the population and dynamically varying decodability of landmark separations within trials (p), the presence of heterogeneous and conjunctive tuning (s), lack of modularity in connectivity between cells with high and low amounts of spatial selectivity (t), and the preservation of cell-to-cell correlations across time within trials and across environments (q). The nonlinearity does affect the distribution of recurrent weights: The distribution of non-diagonal elements in the non-negative network is sparse (excess kurtosis k = 7.8), while it is close to Gaussian for the external and internal map networks with tanh-nonlinearity (k = 0.6 and k = 0.9 respectively; panel u); however, the distributions of eigenvalues of the recurrent weights have similar characteristics for all trained networks (panel v). Structure of the recurrent network: Input neurons encoded noisy velocity (10 neurons) and received external map input (70 neuron), same as the regular external map ANN. Recurrent layer rates were constrained to be non-negative. (**o**) Example tuning curves, same analysis as before. (**p**)Linear decoding of position, displacement from last landmark and landmark separation from ANN activity, same analysis as before. (**q**) ANN maintains pairwise correlation structure across states and environments, same as before. (**r**) Example neurons showing transition from egocentric landmark-relative displacement coding to allocentric location encoding, same analysis as before. (**s**) Example neurons showing conjunctive encoding, same analysis before. (**t**) Distribution of absolute connection strength between and across location-sensitive “place cells” (PCs) and location-insensitive “unselective cells” (UCs), same analysis as before. (**u**) Distribution of non-diagonal recurrent weights for randomly initialized (untrained), external map, internal map, and non-negative network. The k-value measured denotes excess kurtosis, a measure of deviation from Gaussianity (k = 0 for Gaussian distributions). The presence of a nonlinearity constraint on the ANN affects the distribution of recurrent weights: The distribution of non-diagonal elements in the non-negative network is sparse (excess kurtosis k = 7.8), while it is close to Gaussian for the external and internal map networks with tanh-nonlinearity (k = 0.6 and k = 0.9 respectively). (**v**) Scatterplot of real and imaginary part of complex eigenvalues of recurrent weight matrix for randomly initialized (untrained), external map, internal map, and non-negative network. The distributions of eigenvalues of the recurrent weights have similar characteristics for all trained networks.

We report that RSC represents internal spatial hypotheses, sensory inputs and their interpretation and fulfills the requirements for computing and using hypotheses to disambiguate landmark identity using stable recurrent dynamics. Specifically, we found that low-dimensional recurrent dynamics were sufficient to perform spatial reasoning (that is to form, maintain and use hypotheses to disambiguate landmarks over time) in an ANN (Fig. 2 and also see Extended Data Fig. 10 for non-negative ANNs and when no map input was given). We then found that RSC fulfills the requirements for such dynamics, that is, encoding of the required variables (Figs. 1 and 4) with stable low-dimensional (Fig. 3) and smooth dynamics that predicted behavioral outcomes (Fig. 4). Due to the higher trial-to-trial variability and lower number of recorded cells, we do not draw direct connections between specific latent states of the ANN and neural data, as was done in previous studies in primates^2,3,20^ or simpler mouse tasks^19,21^.

We observed that local dynamics in RSC can disambiguate sensory inputs based on internally generated and maintained hypotheses without relying on external context inputs at the time of disambiguation (Fig. 4), indicating that RSC can derive hypotheses over time and combine these hypotheses with accumulating evidence from the integration of self-motion (for example, paths after the first landmark encounter) and sensory stimuli to solve a spatiotemporally extended spatial reasoning task. These results do not argue for RSC as an exclusive locus of such computations. There is evidence for parallel computations, likely at different levels of abstraction, across subcortical^22^ and cortical regions such as PFC^3,23,24^, PPC^25^, LIP^26^ and visual^27,28^ areas. Further, hippocampal circuits contribute to spatial computations beyond representing space by learning environmental topology^29^ and constraining spatial coding using attractor dynamics^19,30,31^ shaped by previous experience^32^. Finally, the landmark disambiguation that we observed probably interacts with lower sensory areas^33^, reward value^9,34^ and action selection computations^21,35^.

The emergence of conjunctive encoding, explicit hypothesis codes and similar roles for dynamics across RSC and the ANN suggests that spatial computations and, by extension, cognitive processing in neocortex may be constrained by simple cost functions^36^, similar to sensory^37^ or motor^38^ computations. The ANN does not employ sampling-based representations, which have been proposed as possible mechanisms for probabilistic computation^39,40^, showing that explicit representation of hypotheses and uncertainty as separate regions in rate space could serve as alternative or supplementary mechanism to sampling.

A key open question is how learning a specific environment, task or behavioral context occurs. We observed that hypothesis coding emerges with task learning (Extended Data Fig. 4). Possible, and not mutually exclusive, mechanisms include: (1) changes of the stable recurrent dynamics in RSC, as is suggested in hippocampal CA1 (ref. ^29^); (2) modification of dynamics by context-specific tonic inputs^3,20^; or (3) changes in how hypotheses and sensory information are encoded and read out while maintaining attractor dynamics that generalize across environments or tasks, as indicated by the maintenance of recurrent structure across tasks in our data (Extended Data Fig. 8) and as has been shown in entorhinal^30^ and motor cortex^38^ and ANNs^41,42^, possibly helped by the high-dimensional mixed nature of RSC representations^43,44^. Further, how such processes are driven by factors such as reward expectation^34^ is an active area of research.

Our findings show that recurrent dynamics in neocortex can simultaneously represent and compute with task and environment-specific multimodal hypotheses in a way that gives appropriate meaning to ambiguous data, possibly serving as a general mechanism for cognitive processes.

## Methods

### Mouse navigation behavior and RSC recordings

#### Drive implants

Lightweight drive implants with 16 movable tetrodes were built as described previously^12^. The tetrodes were arranged in an elongated array of approximately 1,250 × 750 µm, with an average distance between electrodes of 250 µm. Tetrodes were constructed from 12.7-µm nichrome wire (Sandvik–Kanthal, QH PAC polyimide coated) with an automated tetrode twisting machine^45^ and gold-electroplated to an impedance of approximately 300 kΩ.

#### Surgery

Mice (male, C57BL/6 RRID: IMSR_JAX:000664) were aged 8–15 weeks at the time of surgery. Animals were housed in pairs or triples when possible and maintained on a 12-h cycle, at 65–70 °F with ~60% humidity. All experiments were conducted in accordance with the National Institutes of Health guidelines and with the approval of the Committee on Animal Care at the Massachusetts Institute of Technology (MIT). All surgeries were performed under aseptic conditions under stereotaxic guidance. Mice were anesthetized with isofluorane (2% induction, 0.75–1.25% maintenance in 1 l min^−1^ oxygen) and secured in a stereotaxic apparatus. A heating pad was used to maintain body temperature; additional heating was provided until fully recovered. The scalp was shaved, wiped with hair-removal cream and cleaned with iodine solution and alcohol. After intraperitoneal (IP) injection of dexamethasone (4 mg kg^−1^), carprofen (5 mg kg^−1^), subcutaneous injection of slow-release buprenorphine (0.5 mg kg^−1^) and local application of lidocaine, the skull was exposed. The skull was cleaned with ethanol, and a thin base of adhesive cement (C&B Metabond and Ivoclar Vivadent Tetric EvoFlow) was applied. A stainless steel screw was implanted superficially anterior of bregma to serve as electrical ground.

A 3-mm craniotomy was drilled over central midline cortex, a durotomy was performed on one side of the central sinus and tetrode drives^12^ were implanted above RSC, at around anterior–posterior (AP) −1.25 to −2.5 mm and medio–lateral (ML) 0.5 mm, with the long axis of the tetrode array oriented AP and the tetrode array tilted inwards at an angle of ~15–20° and fixed with dental cement. The ground connection on the drive was connected to the ground screw, and the skin around the drive implant was brought over the base layer of adhesive as much as possible to minimize the resulting open wound, sutured and secured with surgical adhesive.

At the time of implant surgery, only two of the tetrodes were extended from the drive to serve as guides during the procedure. All other tetrodes were lowered into superficial layers of cortex within 3 days postsurgery. Mice were given 1 week to recover before the start of recordings.

#### Chronic electrophysiology

After implant surgery, individual tetrodes were lowered over the course of several days until a depth corresponding to layer 5 was reached and spiking activity was evident. Data were acquired with an Open Ephys^46^ ONIX^47^ prototype system at 30 kHz using the Bonsai software^48^ (v.2.2; https://bonsai-rx.org/). The tether connecting the mouse headstage to the acquisition system was routed through a commutator above the arena and was counterbalanced using a segment of flexible rubber tread. Tetrodes were occasionally lowered by small increments of ~50 µm to restore good recording conditions or to ensure sampling of new cells across sessions.

#### Spike sorting

Voltage data from the 16 tetrodes, sampled at 30 kHz were bandpass filtered at 300–6,000 Hz, and a median of the voltage across all channels that were well connected to tetrode contacts was subtracted from each channel to reduce common-mode noise such as licking artifacts.

Spike sorting was then performed per tetrode using the Mountainsort software^49^ (https://github.com/flatironinstitute/mountainsort_examples), and neurons were included for further analysis if they had a noise overlap score <0.05, an isolation score >0.75 (provided by Mountainsort^49^), a clear refractory period (to ensure spikes originated from single neurons), a spike waveform with one peak and a clear asymmetry (to exclude recordings from passing axon segments) and a smooth voltage waveform and ISI (inter spike interval) histogram (to exclude occasional spike candidates driven by electrical noise). Units were not excluded based on firing rates, tuning or any higher order firing properties. The number of simultaneously recorded cells per mouse for the main analyses was as follows. Blackdot, 52,53,54,49; Gothmog, 55,59,52,51,51,85; Nodot, 65,86,72,69; Unnamed1, 67,64; Total, 984. For the entire dataset analyzed in the analysis over learning (Extended Data Fig. 4), a larger number of cells, and of simultaneously recorded cells, were collected, and sessions with <50 cells were included.

#### Histology

To verify the localization of the recording sites (Extended Data Fig. 3), electrolytic lesions were created by passing currents of 20 µA through a subset of tetrodes (roughly four tetrodes per animal) for 30 s each under isoflurane anesthesia, and animals were perfused and brain processed 1 h later. Brains were mounted with 4′,6-diamidino-2-phenylindole and imaged.

#### Behavioral experiment hardware

Behavior was carried out in a circular arena of 50-cm diameter. The floor of the arena was formed by a clear acrylic sheet, under which a diffusion screen and a flat-screen TV was positioned on which visual stimuli were displayed. The circular arena wall was formed by 32 flat black acrylic segments, every other one of which contained an opening for a recessed reward ports, 16 in total. Each reward port contained an optical beam break (880-nm infrared (IR), invisible to mouse) that detected if a mouse was holding its nose in the port, a computer-controlled syringe pump for water reward delivery and a dedicated beeper as a secondary reward indicator. The behavior arena was housed in a soundproof and light-insulated box with no indicators that could allow the mice to establish their heading. Video was acquired by a central overhead camera at 30 Hz using a low level of infrared light at 850 nm and the mouse position was tracked using the oat software^50^ (https://github.com/jonnew/Oat). A custom behavioral control state machine written in Python was triggered every time a new camera frame was acquired, and the position of the animal, time passed and port visits were used to transition the logic of the state machine (Extended Data Fig. 1). For analysis purposes, all behavioral data was resampled to 100 Hz and synchronized to the electrophysiological data.

#### Inactivation of RSC and causal necessity for hypothesis-based computations

For pharmacological inactivation of RSC (Extended Data Fig. 1i–l), four mice were trained on a simplified parametric task that permitted us to causally test the role of RSC in individual recording and inactivation sessions. The task required integration of an allocentric position hypothesis with visual landmarks (Extended Data Fig. 1i,j). After mice learned the task—quantified as reaching a hit rate of above 30% in the simple conditions (high eccentricity; Extended Data Fig. 1j)—they were given access to unrestricted water and implanted following the procedure described for the main experiment but, instead of a chronic drive implant, a removable cap was implanted and two burr holes were prepared above RSC and covered with dental cement (Extended Data Fig. 1k). After recovery from surgery, mice were put back on water restriction over the course of 1 week and reintroduced to the task. Before each experiment, mice were anesthetized briefly with isoflurane, the cap was opened temporarily and the exposed skull was wiped with lidocaine and an injection of either 50 nl of 1 μg ml^−1^ muscimol solution in cortex buffer per side, or the same volume of cortex solution was performed through the existing burr holes. Mice were left to recover from anesthesia for 15 min and tested on the task. Performance was assessed as the hit rate on the first port visit per trial, and confidence level were computed using the Clopper–Pearson method for binomial confidence intervals (CIs) at the 95% level.

#### Behavioral training

After mice had undergone surgery, they were given at least 1 week to recover before water scheduling began. Initially, mice received 3 ml of water per day in the form of 3 g of HydroGel (ClearH2O), which was reduced gradually to 1.0–1.5 g per day. During this period, mice were handled by experimenters and habituated to the arena. Throughout the entire experiment mice were given water rewards for completion of the task and were given additional water to maintain their total water intake at 1.25–1.5 ml.

After initial acclimation to the recording arena over 2 days, mice were trained on the task. Throughout the task we used white circular cues on the floor (referred to as landmarks) of ~30-mm diameter on a black background. These landmarks were the only source of light in the experiment. Mice were run every day or every other day, for a single session of 30 min to 3 h per day. Training progressed in several phases:

1. Initially, mice were trained that circular visual cues on the floor of the arena indicated reward locations. One of the 16 ports was selected randomly as reward port and a cue was shown in front of this port. Visiting an incorrect port resulted in a time out (~1 s initially, increased later), during which the entire arena floor was switched to gray leading to a widespread visual stimulus. Visiting the correct port resulted in an audible beep from the beeper located in the port and around 0.005 ml of water were delivered by the syringe pump. After a reward, a new reward port was chosen randomly, and the landmark was rotated together with the port, effectively performing a rotation of the entire task, and the next trial began. This meant that mice learned to not rely on any cues other than the visual landmark to locate the correct port. Mice usually completed this phase in by day 4.
2. We then introduced a new task phase, referred to in the text as ‘dot-hunting’ task: after each reward, the landmark disappeared and instead a blinking dot was shown in a random location in the arena. If the mouse walked over that dot, it disappeared and either a new dot in a new random location appeared, repeating the process, or the next trial was initiated. The number of required dots–chases was sampled uniformly from a range and was increased to six to eight by the time recordings began, and the last dot was always positioned at the arena center. This task phase served to obfuscate the rotation of the task. Data acquired during this task phase were used during spike sorting but were not part of the main dataset in which we analyzed hypothesis representation. We analyze this task phase separately in Fig. 3c and Extended Data Figs. 3h,i, 4 and 8. Mice learned this task phase, with six to eight dots, by day 7 on average.
3. Throughout phases 1 and 2, we progressively introduced a requirement for the mice to hold their snouts in the reward port for increasing durations to trigger a reward or time out. For each port visit, the required duration was drawn randomly from a uniform distribution, so on any given trial the mice did not know when exactly to expect to know the outcome of the port visit. Initially, this hold time was 500 ms, and the time range was slowly increased throughout training, depending on animal performance. By the time recordings began, a range of around 4–6 s was used. Mice were able to tolerate this holding time by day 20 on average.
4. Next, we introduced an identical second landmark at a nonrewarded port. Initially, the two landmarks were set two ports apart (for example, ports 1 and 3), and this distance was progressively increased to four or five ports. As before, the rewarded port and landmarks were rotated randomly after each trial, but their relative positions remained stable. Visiting the reward port at the incorrect, ‘a’ landmark (and holding there for the required duration) was handled identically to visits to any other nonreward port and triggered the same time out. As a result, mice learned to visit the ‘b’ port. Mice learned to make an initial distinction between the ports approximately by day 14–16. In one mouse, we maintained this training phase until overall task performance was significant over entire sessions (Extended Data Fig. 1f), but we noticed that the mouse had trouble consistently relearning the next task phase. We therefore transitioned subsequent mice to the next phases before a stable behavior was established.
5. After the mice started learning to visit the port at the ‘b’ landmark, we introduced a view distance limitation that made landmarks invisible from far away: the mouse’s position was tracked at 30 Hz and, for each landmark, its brightness was modulated in real time as a function of the mouse’s distance from it. The visibility was 0 for distances above a threshold, 1 for distances below a second threshold and transitioned linearly between the two values. For clarity, we draw only the first threshold where landmarks initially become visible in the illustrations. The second threshold was typically set to about 50% of the first, leading to a gradual brightening, but in the otherwise totally dark arena, almost any values >1 are clearly visible. Initially, thresholds were set so that both landmarks were visible from the arena center (~20 cm); they were then reduced progressively to values where, at any one time, only one of the landmarks was visible to the mouse (~10 cm). At this stage, mice that encounter a landmark after a new trial starts have no way of knowing whether this is the rewarded or nonrewarded landmark, unless they infer landmark identity via path integration (See Fig. 2e right or Extended Data Fig. 2e). Recordings began when mice were able to complete 100 trials per hour at a hit/miss rate >1. Mice reached this criterion level on average by total day 30–40 of training.

#### Statistics and reproducibility

Statistical tests were carried out in Matlab (Mathworks, v.2019) using built-in functions. Unless stated otherwise, CIs were computed at a 95% level using bootstrap, and *P* values were computed using a Mann–Whitney *U* test or Wilcoxon signed-rank test. In figures, significance values are indicated as nonsignificant (NS) (*P* > 0.05), *(*P* ≤ 0.05), **(*P* ≤ 0.01) or ***(*P* ≤ 0.001). No statistical method was used to predetermine sample sizes.

#### Behavior analysis

Recording sessions were included once mice performed the task well enough to achieve a session average hit/miss ratio >1, indicating that mice could infer the correct port between the ‘a’ and ‘b’ landmarks (a correct rate of >1/16 would indicate that they can associate landmarks with rewarded ports, but not that they can infer landmark identity). Because landmarks are visible sequentially only after full training, a ratio >1 shows that mice employed a memory based strategy where they used a previous hypothesis derived from seeing or not seeing the first landmark, together with path integration, to infer the identity of the second landmark they encounter. Only sessions with at least 50 recorded single neurons, and with at least 50 min of task performance were included. This yielded 16 sessions from four mice. For some analyses, particularly for analyses where trajectories of the mice were matched across trial types to control for potential motor and sensory confounds, additional selection criteria were applied yielding a lower number of sessions that could be used, this is stated for the respective analyses. For plots of the learning rates, we included trials where mice encountered their first landmark after 20 s or faster to exclude periods where mice were not engaged.

#### Behavioral epochs

For analysis, each trial was split into epochs: the time between the onset of a trial (right after the mouse completes the preceding reinitialization procedure, and finds itself at the center of the arena, unsure of its orientation relative to the currently invisible landmarks) and the onset of the reward (the first time the mouse could know whether it reached the correct port, other than by process of elimination after visiting all other ports) was split up based on the amount of information the mouse could have accumulated: the initial state when mice had not seen any landmark was labeled ‘LM0,’ time after the first landmark encounter was labelled ‘LM1,’ and after the second encounter as ‘LM2’. The timepoints when landmarks became visible and the mouse transitioned from LM0 to LM1 or from LM1 to LM2, referred to as ‘landmark encounters’ were defined as the timepoint when landmark visibility exceeded 50%.

For analyses of the correlation of neural state and eventual behavioral outcomes, each second landmark encounter was further categorized as whether it occurred at the ‘a’ or ‘b’ landmark. For behavioral analyses in Fig. 4d, trials were further categorized by whether they led to a correct port visit or to a incorrect visit and a time out.

#### Similarity of spatial tuning across conditions

Changes in spatial tuning in individual RSC neurons as mice encounter successive landmarks (Fig. 1f) was quantified by the Euclidian distance of their spatial tuning profiles (in an 8 × 8 map, resulting in a 64-element vector, for each comparison nonvisited ties were omitted). As an internal control, distance between tuning profiles within condition and across condition were compared using nonoverlapping 1-min segments. The control levels are different between the cases because the amount of data per session, reliability of firing, and so on, is not constant, and each control is valid only for its test data. For each comparison (LM1 versus LM2 and LM0 versus LM1), the split spatial tuning maps were compared either within the conditions, for example, within LM1 and within LM2, and compared with distances between LM1 and LM2 maps.

#### Neural decoding of mouse position

All decoding analyses were performed on the entire neural population with no preselection. To decode the mouse position from RSC firing rates, neural firing rates were first low-pass filtered at 1 Hz with a single-pole Butterworth filter. The resulting firing rate time series were used to predict the mouse position as 100 categorical variables forming a 10 × 10 bin grid (bin width = 50 mm). The network was made up of a single long short-term memory (LSTM) layer with 20 units, and a fully connected layer into a softmax output into the 100 possible output categories. For analyses of intermediate information content of the decoder, the network input into the final softmax layer was analyzed.

Decoding was reinitialized for each trial. For each decoded trial, all other trials served as training set. For analysis of how the neural coding of position was dependent on the landmark state of the mouse (Extended Data Fig. 2a), the same analysis was repeated with training and testing data further divided by landmark state. For analysis of the decoding performance, the output likelihood from the decoder was evaluated at the mouse’s true position for all positions that were shared across conditions for this session. Statistical analysis was then performed on a per session average likelihood (not weighted by number of trials per session).

#### Neural decoding of landmark state

For the analysis of landmark state (Fig. 1e), trials with at least 0.5 s of data from all three states were used (16 sessions, 486 total trials) and individual trials were held out from training for decoding. Firing rates were low-pass filtered with a causal single-pole Butterworth filter at 0.05 Hz, and landmark state (0, 1 or 2) was decoded independently for each timepoint using a categorical linear decoder (dummy variable coding, (*N*_neurons _+ 1) × 3 parameters), or a neural network with no recurrence, using a single 20-unit layer receiving instantaneous firing rates, into a six unit layer and into three softmax outputs. Training data were balanced across conditions. For related analyses of hypothesis state decoding, see also Fig. 4 and Extended Data Fig. 9j, where we decode form position-matched timepoints to account for location, motor and visual confounds, and Extended Data Fig. 7 where we match for position.

#### Analysis of landmark ‘nonencounters’

To show that mice can gain information by not encountering a landmark (as is shown, for example, by the ANN example in Fig. 2), we analyzed cases where the mouse first encounters a landmark, and then, in the LM1 state, encounters the position where another landmark could be, but fails to see one. We note that this analysis has unavoidable confounds, as in one condition the mouse gets salient visual input, in the other it does not. We consequently ignored these cases in the main analysis, and instead concentrated on cases where visual input was matched, but previous hypotheses differ (Fig. 4). We analyze these nonencounters by decoding the associated state change with the same method as in ‘Neural decoding of landmark state,’ but with nonbalanced conditions, due to the lower trial count, and analysis of the prediction around the 0-s point.

#### Dimensionality analysis

PCA was performed by first computing the covariance matrices of the low-pass filtered (as before) firing rates, and plotting their eigenvalue spectra, normalized by sum (Extended Data Fig. 8c). Each scaled eigenvalue corresponds to a proportion of explained variance. Spectra are plotted together with a control spectrum computed from covariances of randomly shuffled data. For a description of the method used to compute the correlation dimension of RSC rates (Extended Data Fig. 8d), see the heading ‘Correlation dimension’ in the section about ANN methods below.

#### Prediction of firing rates across RSC population

For quantification of the independence of individual RSC neurons from the surrounding RSC population (Extended Data Fig. 8f,g), the firing rates of each neuron were predicted from those of all other neurons using linear regression. Rates were first filtered at 0.01–0.5 Hz with a third-order Butterworth filter, and subsampled to 3.3 Hz. Each neuron’s rate was predicted with L_1_ regularized linear regression^51^ (${\rm{lasso}},\lambda \approx 0.0001$) from the rates of all other neurons and preceding firing rates using eight lags (~0.2.5 s). Goodness of fit was quantified as the proportion of variance explained, ${R}^{2}=1-{\sum }_{i}{({Y}_{i}-{Y}_{i}^{{\rm{pred}}})}^{2}/{\sum }_{i}{\left({Y}_{i}-\bar{Y}\right)}^{2}$. Predictions were computed both within condition (LM1, LM2 and dot-hunting phase), as well as across conditions, where the model was fit using coefficients determined from the other conditions.

#### Computation of firing rate distribution entropies

Entropies of empirical firing rate distributions were computed in bits according to their Shannon entropy, $H\left(X\right)=-{\sum }_{i=1}^{n}P\left({x}_{i}\right){\log }_{2}P({x}_{i})$, relative to a uniform histogram of the same size, $\hat{H}\left(X\right)=-\left(H\left(X\right)-H\left({\rm{uniform}}\right)\right)$. In cases where zeros appeared, a small offset term <<1 was added and all histograms were normalized to a sum of 1. For example, $\hat{H}\left(\left[1,0\right]\right)=\hat{H}\left(\left[1,1,0,0\right]\right)=1\,{\rm{bit}}$, and $\hat{H}\left(\left[1,1,1,1.3\right]\right)\cong 0.01\,{\rm{bit}}$. For the analysis in Fig. 1g, a 8 × 8 grid was used for spatial coding, and three bins for the state coding. Although the 8 × 8 grid is coarse enough to allow accurate capture of the spatial firing rate profile even for low-rate cells, the resulting estimates could be minimally affected by firing rate differences between neurons.

#### Trial-to-trial variance of firing rates conditioned on position

For analysis of whether partial hypothesis representation in the LM1 state corresponds to trial-by-trial changes in firing rates, evident in bimodal firing rate histograms, histograms of hidden unit firing rates of the ANN, conditioned on binned 1D position are displayed (Extended Data Fig. 7a). Data are from Experiment configuration 2 (‘Overview over experiment configurations used with ANNs’). Tuning curves were calculated using 20 bins of location/displacements and normalized individually for each neuron. The first timestep in each trial and timesteps with nonzero landmark input were excluded from the analysis. For histograms, each condition was binned in 100 column bins and neuron rates in ten row bins. Histograms were normalized to equal sum per column. For analysis of RSC firing rates (Extended Data Fig. 7b–d), we did not observe bimodal rate distributions and instead quantified the dispersion of the rate distributions according to their entropy: firing rates were low-pass filtered at 0.5 Hz to bring them into the timescale of navigation behavior, and firing rate histograms were computed with eight bins spanning from each neurons lowest to highest firing rate per neuron, for each spatial bin in a 4 × 4 grid. Because the computation of histogram entropy is biased by the number of samples, for each spatial bin, the same number of timepoints were used for the LM1 and LM2 conditions. The dispersion of the firing rate distribution was then computed as average entropies per cell across all space bin, and compared across the two conditions.

#### Analysis of encoding of angular position and displacement from last seen landmark

Firing rate profiles were analyzed in two reference frames, that is, global angle of the mouse in the arena, and relative angle to the last visible landmark. Only timepoints from the foraging state where the distance of mouse from the center of the arena exceeded 70% of the arena diameter were included. Timepoints from the LM1 and LM2 conditions were subsampled to yield matched number of timepoints. Firing rates were analyzed in a −π to π range in six bins by computing their entropy as described before.

#### Pairwise correlation of firing rates

Recordings were split into LM[0,1,2] states as before, firing rates were low-pass filtered at 1 Hz, and the Pearson correlation coefficient between each pair of neurons was computed. For display purposes, the neurons were reordered by first computing the matrix for the LM1 state, applying hierarchical clustering^52^, and the resulting reordering was applied to both LM1 and LM2 conditions. This reordering has no impact on any further analyses. For summary statistics, we computed the correlation of correlations for each session. We observed no systematic change in the results as a function of the low-pass cutoff frequency, see Extended Data Fig. 8e for a comparison of a 1-Hz versus a 5-Hz cutoff.

#### Low-dimensional embedding of neural activity

Neural firing rates were bandpass filtered as before, and an initial smoothing and dimensionality reduction step was performed by training a small LSTM with a single layer of 30 units to decode the mouse position. The hidden unit activations were then embedded in 3D space with the isomap algorithm^53^, using the Toolbox for Dimensionality Reduction by Laurens van der Maaten^54^.

#### Analysis of speed of neural state evolution

For quantification of how fast the neural state evolves, the firing rates of the entire population were computed by low-pass filtering the spike trains at 1 Hz (third-order Butterworth filter), and the speed of the five largest principal components of the resulting vector in Hz s^−1^ were related to the running speed of the mouse (m s^−1^, also low-pass filtered at 1 Hz) or the change in landmark brightness (percent per second) (Extended Data Fig. 9a). Data were binned in 30 bins from 0 to 0.5 m s^−1^ and ten bins from 0.5 to 2 m s^−1^ for running speed and ten bins from −50 to 50% and ten bins for ±50–200%. CIs were computed by treating median data from each session as independent samples.

#### Analysis of context-encoding in RSC across similar motor and sensory states

To study the encoding of context with minimal sensory and motor confounds (Fig. 4 and Extended Data Fig. 9), we split the appearances of the second landmark into two groups depending on whether the second landmark is ‘a’ or ‘b,’ as described in the main text. We then selected subsets of trials manually where egocentric paths just before the appearance of the second landmark are matched across the two groups. Figure 4a shows an example of such matched approach paths/trials. Sessions in which at least 16 trials could be matched were used for these analyses, yielding a total of 133 trials from six sessions (per session, 16, 23, 24, 24, 25 and 21). For each session, all of these trials were aligned to the time when the second landmark became visible, yielding a set of time ranges where the animals experienced similar visual inputs, performed similar locomotion behavior but potentially encoded different previous experience leading them to subsequently disambiguate the perceptually identical second landmark as ‘a’ or ‘b.’

To test whether there was consistent encoding of this context in RSC, we then compared the distances across these groups in 3D neural activity space (‘Low-dimensional embedding of neural activity’) to distances within the groups (Fig. 4b and Extended Data Fig. 9). This test was performed at the point where the second landmark became visible to assess encoding of previous context, as well as 200 ms afterwards to assess how the identity of the (now visible) landmark affects encoding in RSC.

#### Analysis of smooth neural trajectories across sessions

To assess whether neural trajectories were determined by population dynamics that were stable across trials and could therefore serve as substrate for the computation performed by the mice, we tested whether neural trajectories behaved consistent with a laminar flow regime where neighboring particles (in our case, neural firing rate vectors) remain neighbors for a significant amount of time, or whether they decorrelate quickly (Fig. 4c and Extended Data Fig. 9e,f). To assess temporal dynamics of the neural spiking without imposing any smoothing, we investigated raw spike counts in 750-ms windows for this analysis. For each session, an initial set of pairwise high-dimensional distances in spike counts between the trials with egocentrically similar paths (‘Analysis of context-encoding in RSC across similar motor and sensory states’) was computed from the last 750 ms preceding the appearance of the second landmark. These distances were then correlated with those in a second sliding window; Extended Data Fig. 9f). An offset of 0 s was defined as the point where both windows stopped overlapping. The correlation coefficient *R* was then computed for increasing window offset up to 2 s. Summary statistics were computed across sessions by first shifting each session individually by its 95% level for *R* (from a shuffled control which removed the relationship between cells) which results in the summary plot showing a highest value for *R* of ~0.8 even for offsets where the windows fully overlap and the uncorrected *R* value is 1. Because of this offset, the null level for each trial is now at *R* = 0. We then computed the CIs for the group by bootstrap relative to this level.

#### Decoding of low-dimensional task-relevant states from RSC activity

To illustrate the joint encoding of position and task states (as sketched conceptually in Fig. 4f) using neural data, we decoded the hypothesis state, as well as *x*/*y* position from firing rates (Fig. 4e). Individual trials were held out as test set, an ANN was trained on the remaining trials and the resulting predictions in the test trial were plotted with hypothesis state in *z* and *x*/*y* in *x*/*y* dimensions. True LM0, 1a and 1b states were indicated with same colors as throughout the figure. Rates were low-pass-filtered with a causal third-order Butterworth filter at 0.5 Hz to bring rates into the behavioral timescale. For position decoding, the network architecture was filtered rates > 20-unit LSTM layer > 15-unit LSTM > 6-unit LSTM > 2 element regression output (mouse *x*/*y* position). For hypothesis states, rates > 10-unit LSTM > single regression layer, with LM0 encoded at 0, and LM1a and b as −1 and +1, respectively. This analysis was not used to make statistical statements. Instead, we tested *x*/*y* encoding in Fig. 1, and hypothesis encoding in Fig. 4 and Extended Data Fig. 9 with statistical methods.

#### Analysis of direction of neural trajectories

To further test whether neural trajectories were determined by population dynamics that were stable across trials, and were independent of the interpretation of the second (locally ambiguous) landmark, we tested whether neural activity evolved in similar directions across trials if it started close together in 3D neural activity space (‘Low-dimensional embedding of neural activity’) (Extended Data Fig. 9g,h). We therefore looked at neural trajectories within the motor and sensory-matched LM2 approaches where the neural state at the point where the second landmark became visible started neurally close to other trials from the opposing class. For example, for an LM2_a_ trial, we examined whether this trial might follow other close-by LM2_b_ trials. We computed neural proximity in the 3D neural embedding (see above) and defined close-by trials as ones that were within 1 a.u. in Euclidean distance in the isomap embedding around the time when the second landmark became visible, yielding a total of 42 out of 79 trials with close neighbors from opposing classes from the five sessions (one session was excluded because the neural activity in the relevant time ranges was collapsed onto a point in the LSTM embedding). As a control, we also selected corresponding neurally furthest points. Similarity of neural evolution was then quantified as the angular difference between the trials in (3D) LSTM space over time, to assess coevolution independently of the initial selection by distance. Significance was computed by bootstrap across trials versus random alignments corresponding to a 90-degree difference.

#### Behavior prediction

For the behavior prediction analysis, sessions with at least five correct and incorrect port visits after the second landmark visit were used (*N* = 11) and an equal number of hit and miss trials (outcome of next port visit is a time out or a correct) were selected, leading to a chance prediction level of 0.5. The spike rates from the 5 s preceding the second landmark becoming visible, binned into 1-s bins, were used to predict the behavioral outcome with a binary classification decision tree with a minimum leaf size of six, previously determined using cross-validation. Predictions for each trial were fit using all other trials.

#### Specificity of landmark encounter coding to the foraging task

We trained a decoder to predict either the number of encountered dots in the main task, or in the dot-hunting task. These tasks were interleaved, and the same neurons were used (Extended Data Fig. 4). Train and test sets were split by trial, and decoding was performed with a regression tree on low-pass-filtered firing rates as before, performance was quantified as mean error on the number of landmarks. Only the first two landmarks were predicted in the dot-hunting task to allow use of the same classifier across both. Decoding performance was compared between the within-class (for example, decode main task encounters with decoder trained on other trials in the main task) and cross-class (for example, decode dot-hunting from decoder trained on the main task, and so on).

#### Analysis of neural coding as a function of task performance

To test whether the encoding of hypothesis states in RSC is specific to task performance, we analyzed a larger number of sessions from the entire period during which two landmarks with local visibility were used (92 recording sessions in total) (Extended Data Fig. 4). We analyzed the effect of task performance on the behavior prediction analysis (as described above; Extended Data Fig. 4). We also analyzed the more general decoding of landmark encounter count (same method as in ‘Specificity of landmark encounter coding to the foraging task’; Fig. 1) in all of the 92 sessions with two landmarks, and correlated decoding performance with task performance on a per session level. As an additional control, we performed the same analysis on the number of dots encountered in the interleaved dot-hunting task. For all of these analyses, we used an analogous method as for the nonbehavior-correlated analyses.

#### Correlation dimension in RSC

For details of the calculation of the correlation dimension for RSC data, see ‘Correlation dimension.’

### Artificial neural networks

We chose a simple recurrent neural network as one of the simplest architectures that can learn to maintain state over time. Unless stated in the text, the default architecture consisted of rate neurons with an input layer into 128 hidden recurrent units (tan*h* nonlinearity) into 80 output neurons, trained on random velocity trajectories in random environments of up to four landmarks (see ‘Network architecture and training’ for details). For the analyses in the main text, landmark inputs were relayed to the ANN as a map that encoded their relative position but not identity (‘external map’ ANN, 80 input neurons). The findings were replicated with an ANN that received only binary landmark presence input (‘internal map’ ANN, 11 input neurons) and non-negative ANNs (Extended Data Fig. 10), on a subset of environments. The ANN serves to establish whether and how recurrent systems could solve the task, and we make no connections between the circuitry of RSC and the connections in the ANN.

A simulated animal runs with varying velocity in a circular environment starting from a random unknown position and eventually infers its position using noisy velocity information and two, three or four indistinguishable landmarks. A trial consists of a fixed duration of exploration in a fixed environment, starting from an unknown starting location; the environment can change between trials. Environments are generated by randomly drawing a constellation of two to four landmarks, and the network must generalizably localize in any of these environments when supplied with its map. The network must adjust its spatial inference computations on the basis of the configurations of the different environments, without changing its weights; thus, the adjustments must be dynamic. In the internal map scheme (Extended Data Fig. 10a–m), an input cell simply encodes by its activation whether the animal is at any landmark; it does not specify the location of the landmark, the identity of the environment, or the spatial configuration of the various landmarks in the environment. The task in the internal map scheme is substantially harder, since the network must infer the configuration of landmarks in the environment purely from the time sequence of landmark visits, while simultaneously localizing itself within the environment. Information about the maps must be acquired and stored within the network. To make the task tractable, we limit training and testing in the internal map setting to four specific environments.

In the external map task (Figs. 2 and 3 and Extended Data Figs. 2 and 5–7), landmark locations were random and the set of locations (map) were provided to the network, whereas in the internal map task (Extended Data Fig. 10a–m) one of four landmark configurations was used, but the maps were not provided to the network. Landmarks could be observed only for a short distance. A three-layer network with a recurrent hidden layer was trained to infer location. Velocity and landmark encounter information were encoded in the input layer, and all weights of the network were trained. The training target for the output layer was activation of a unit with von Mises tuning and preferred location matching the true location.

Network performance was compared with a number of alternative algorithms: path integration plus correction integrated the noisy velocity information starting from an initial location guess and corrected this estimate by a reset to the coordinates of the nearest landmark when a landmark was encountered. Particle filters approximated sequential Bayesian inference given the available velocity and landmark information, with each particle capturing a location hypothesis whose posterior probability is given by an associated weight. Particle locations are updated using velocity information and particles are reweighted after landmark encounters. The enhanced particle filter also reweights particles when a landmark is expected but not encountered, thus can infer location not only from the presence but also from the absence of landmarks. The output and hidden representations of the trained network were evaluated in a variety of conditions involving both random and fixed landmark locations and trajectories with random and fixed velocities.

#### Definition of environments and trajectories

The task is defined by a simulated animal moving along a circular track of radius 0.5 m for 10 s. The animal starts at a random, unknown position along the circle at rest and starts running along a trajectory at nonconstant velocity. A trajectory is sampled every *d*_*t*_ = 0.1 s in the following way: at each time *t*, acceleration *a*_*t*_ is sampled from a zero-mean Gaussian distribution with s.d. *σ*_a_ = π/4 m s^−2^ that is truncated if |*a*_*t*_ | > π/2 m s^−2^. Acceleration is integrated to obtain the velocity *v*_*t*_ and truncated if |*v*_*t*_ | > *v*_max_ = π/2 m s^−1^. The actual location on the track is the integral of this velocity.

In a trial of the external map task, the locations of *K* = 2, 3 or 4 indistinguishable landmarks were determined sequentially: the first landmark was sampled from a uniform random distribution on the circle, with subsequent landmarks also sampled from a uniform random distribution but subject to the condition that the minimum angular distance from any previously sampled landmark is at least *δ* = π/9 rad.

The internal map task involved four environments, each with a unique configuration of landmarks: two environments had two landmarks, one had three and the last had four. Landmark locations in the four environments were chosen so that pairwise angular distances were sufficiently unique to allow the inference of environment identity. Landmark coordinates in environment *e*_*i*_ were given by: *e*_1_ = {0, 2π/3} rad, *e*_2_ = {1.9562, 3.7471} rad, *e*_3_ = {0.2641, 1.2920, 3.7243} rad and *e*_4_ = {3.0511, 3.8347, 5.1625, 5.7165} rad.

#### Experiment configurations used with ANNs

After training, the networks were evaluated in different testing configurations that each consisted of a distribution over landmark configurations and trajectories:

##### Experiment configuration 1

Training distribution: this test set was generated exactly as in the training set, as described in ‘Definition of environments and trajectories’.

##### Experiment configuration 2

Fixed landmarks, random trajectories: the landmark configuration was given by two landmarks located at *e* = {0, 2π/3}, the trajectories were sampled in an identical way as in the training distribution. Note that this landmark configuration corresponds to the first environment in the internal map task.

##### Experiment configuration 3

Fixed landmarks, constant velocity trajectories: the landmark configuration was given by two landmarks located at *e* = {0, 2π/3} and the trajectories were given by constant velocity trajectories with |*v*_*t*_ | = *v*_max_/2. The initial position and the direction of the trajectory was random.

##### Experiment configuration 4

Two variable landmarks, constant velocity trajectory: the landmark configuration was given by two landmarks located at *e* = {0, 2π/3 + απ/3}, where α ϵ [0, 1]. The trajectories were given by constant velocity trajectories with |*v*_*t*_ | = *v*_max_/2 and the initial position and the direction of the trajectory was random.

##### Experiment configuration 5

Two environments, random trajectories: the landmark configuration was given by either *e*_1_ or *e*_2_ of the internal map task, trajectories are random.

#### Landmark observation

The animal is considered to have encountered a landmark if it approached within *d*_min_ = *v*_max_ × *dt/*2 = π/40 m^−2^ = π/20 rad. This threshold is large enough to prevent an animal from ‘missing’ a landmark even if it is running at maximum velocity. This ‘visibility radius’ is smaller than the one we used for the mouse behavior experiments (Fig. 1). In the ANN experiments, landmark encounters were therefore roughly coincident with the agent’s position coinciding with the landmark, whereas in the mouse data, landmark encounters occur a significant distance away from the landmark, when it becomes visible (for example, Fig. 4a). In the same way as in the mouse behavior analysis, hovering around the same landmark or approaching the same landmark consecutively would trigger a landmark encounter only at the first approach; a new encounter was triggered only if the animal approached an landmark different from the previous one, equivalent to the definition used in the analysis of mouse behavior. Also, only trials in which the animal encountered at least two different landmarks were included.

#### Sensory noise

The largest sources of uncertainty in the tasks were the unknown starting position and the indistinguishability of the landmarks. In addition, we assumed that the velocity information and the landmark location memory (in the external map scenario) were corrupted by noise. At each timestep of size *d*_*t*_ = 0.1, the velocity input to the network corresponded to the true displacement *vd*_*t*_ corrupted by zero-mean Gaussian noise of standard deviation *σ*_v _= *v*_*max*_*d*_*t*_/10. In the external map task, the landmark map provided to the network and particle filter was corrupted by zero-mean Gaussian noise with standard deviation *σ*_*l*_ = π/50 rad, without changing the relative landmark positions: The map was coherently slightly rotated at a landmark encounter, and the rotation was sampled independently at each landmark encounter.

#### ANN preferred firing at landmark locations

This analysis was performed by evaluating the network of the external map task on the experiment configuration 1 of the internal map task (Extended Data Fig. 2c). First, location tuning curves were determined after the second landmark encounter using 5,000 trials from distribution 1 and using 50 location bins. Tuning curves were calculated separately for each of the four environment of the internal map task. Preferred location was determined to be the location corresponding to the tuning curve maximum. The density of preferred locations smaller than distance *d*_min_ away from a landmark was then compared with the density of preferred locations further away from landmarks.

#### Network architecture and training

The network consisted of three layers of rate neurons with input-to-hidden, hidden-to-hidden and hidden-to-output weights. All weights were trained.

##### Network input

The input layer consisted of 80 neurons in the external map case and 11 neurons in the internal map case. Ten neurons coded for velocity corrupted by noise (noise as described above). The velocity neurons had a minimum firing rate between 0 and 0.2 and a maximum firing rate between 0.8 and 1 in arbitrary units, and within this output range coded linearly for the whole range of velocity between −*v*_max_ and *v*_max_. Negative and positive velocity here corresponds to CW and CCW travel, respectively.

The remaining neurons (70 in the external map case and 1 in the internal map case) coded for landmark input and were activated only at the timestep of, and up to, three timesteps after a landmark encounter. In the external map case, the landmark input simultaneously encoded the locations of all landmarks in the environment, thus supplying a map of the environment, but contained no information about which LM was currently encountered. The LM neurons had von Mises tuning with preferred locations *x*_*j*_ = (*j* − 1) × 2π/70 rad, *j* = 1…70, that tiled the circle equally. Given *n* landmarks at locations *l*_*i*_, *i* = 1…*n*, the firing rate of the *j*-th landmark input neuron was given by

$${r}_{j}=\mathop{\sum }\limits_{i}\exp \left(\frac{\cos \left({x}_{j}-\widetilde{{l}_{i}}\right)-1}{2{\sigma }_{w}^{2}}\right),$$

where $\widetilde{{l}_{i}}\sim N\left({l}_{i},{\sigma }_{l}^{2}\right)$ is the noise-corrupted landmark coordinate (‘Sensory noise’). This mixture of von Mises activation hills produces the pattern depicted as the ‘map’ input in Extended Data Fig. 5a.

In the internal map case (Extended Data Fig. 10a–m), the landmark input neuron consisted of a single binary neuron that responded for four timesteps with activation 1 in arbitrary units whenever a landmark was encountered. This input encoded neither environment identity nor landmark location.

##### Hidden layer

The hidden layer consisted of 128 recurrently connected neurons. The activation *h*_*t*_ of hidden layer neurons at timestep *t* was determined by *h*_*t*_ = tan*h*(*W*_*x*_x_*t*_ + *W*_*h*_*h*_*t*_ − 1 + *b*), where *x*_*t*_ are the activations of input neurons at timestep t, *W*_*x*_ are the input-to-hidden weights, *W*_*h*_ are the hidden-to-hidden weights and *b* are the biases of hidden neurons. The nonlinearity should be considered as an effective nonlinearity at long times; since the timestep *d*_*t*_ = 0.1 s was large compared with a typical membrane time constant (*τ* ≈ 0.02 s), we did not include an explicit leak term.

##### Hidden layer (non-negative network)

In the non-negative network (Extended Data Fig. 10n–t), the recurrent activation was determined by *h*_*t*_ = tan*h*([*W*_*x*_*x*_*t*_ + W_*h*_h_*t*−1_ + *b*]_+_), where [*u*]_+_ denotes rectification.

##### Output layer

The output layer consisted of a population of 70 neurons with activity *o*_*t*_ given by *o*_*t*_ = tan*h*(*W*_*o*_*h*_*t*_ + *b*_*o*_), where *W*_*o*_ are the output weights and *b*_*o*_ the biases of the output neurons.

##### Network training

The training targets of the output layer were place cells with von Mises tuning of width *σ*_*o*_ = π/6 rad to the true location *y*_*t*_,

$${\widetilde{o}}_{\alpha ,t}=\exp \left(\frac{\cos \left({z}_{a}-{y}_{t}\right)-1}{2{\sigma }_{o}^{2}}\right),$$

where *z*_*α*_, *α* = 1…70 are the equally spaced preferred locations of each training target.

The network was trained by stochastic gradient descent using the Adam algorithm^55^, to minimize the average square error between output *o*_*t*_ and training targets ${\tilde{{o}}_{t}}$, with the average taken over neurons, time within each trial and trials. The gradients were clipped to 100. The training set consisted of 10^6^ independently generated trials. During training, performance was monitored on a validation set of 1,000 independent trials and network parameters with the smallest validation error were selected. All results were cross-validated on a separate set of test trials to ensure that the network generalized across new random trajectories and/or landmark configurations.

##### Network location estimate

Given the activity of the output layer at time *t*, we define the network location estimate for that time to equal the preferred location (the preferred location was set over training) of the most active output neuron:

$${\hat{{y}_{t}}}={z}_{{\hat{\alpha }}_{t}},{\hat{\alpha }}_{t}=\text{argma}{\text{x}}_{\alpha }{o}_{\alpha ,t}$$

#### Performance comparisons

In Fig. 2b, we compared the performance of the network in the external map task with a number of alternative algorithms. To ensure a fair comparison, we make sure that each alternative algorithm has access to exactly the same information as the network: the landmark identities are indistinguishable and both velocity and landmark location information are corrupted by the same small amount of sensory noise. Error statistics were computed from 5,000 trials.

##### Path integration and correction

This algorithm implements path integration and landmark correction using a single location estimate, similar to what is implemented in hand-designed continuous attractor networks that implement resets at boundaries or other landmarks^15,16,56,57^. The algorithm starts with an initial location estimate at *y* = 0 (despite the true initial location being random and unknown), and integrates the noise-corrupted velocity signal to obtain location. At each landmark encounter, the algorithm corrects its location estimate to equal the coordinates of the landmark nearest to its current estimate.

##### Basic particle filter

Particle filters implement approximate sequential Bayesian inference using a sampling-based representation of the posterior distribution. Here, the posterior distribution over location at each timepoint is represented using a cloud of weighted particles, each of which encodes through its weights a belief, or estimated probability, of being at a certain location. In the beginning of the trial, *N*_*p*_ = 1,000 particles are sampled from a uniform distribution along the circle and weighted equally. In the prediction step, particles are propagated independently using a random walk whose mean is the noise-corrupted velocity update and whose s.d. is the velocity noise *σ*_*v*_. In the absence of a landmark encounter, particle weights remain unchanged and the particle cloud diffuses. If a landmark is encountered, the importance weights *w*_*t,β*_ of particles *β* = 1…*N*_*p*_ are multiplied by

$${w}_{t,\beta }\propto {w}_{t-1,\beta }\bullet \mathop{\sum }\limits_{i}\exp \left(\frac{\cos \left({y}_{t,\beta }-{\widetilde{l}}_{i}\right)-1}{2{\sigma }_{l}^{2}}\right)$$

where *y*_*t,β*_ are the current estimates of the particles, and the weights are subsequently normalized such that ${\sum }_{\beta }{w}_{t,\beta }^{2}=1$. If the effective number of particles becomes too small, that is, ${N}_{\text{eff}}=1/{\sum }_{\beta }{w}_{t,\beta }^{2} < {N}_{p}/5$, the particles are resampled using low variance sampling^58^ and the weights equalized. This resampling step both allows for better coverage of probabilities and permits the particle cloud to sharpen again. The particle filter estimate at a given timepoint is given by the weighted circular mean ${\hat{y}}_{t}={{\arg }}({\sum }_{\beta }{w}_{t,\beta }\exp (i{y}_{t,\beta }))$ of the particle locations. In addition, we also calculate the circular variance as $\mathrm{var}\left({y}_{t}\right)=1-\left|{\sum }_{\beta }{w}_{t,\beta }\exp \left(i{y}_{t,\beta }\right)\right|$.

##### Enhanced particle filter

This particle filter has identical initialization, prediction step and weight update at landmark encounters as the basic particle filter and proceeds in exactly the same way until the first landmark encounter. Subsequently, the enhanced particle filter can also use the absence of expected landmark encounters to narrow down its location posterior, similar to the network’s ability shown in Extended Data Fig. 5. This is implemented in the following way: if a particle comes within the observation threshold *δ* of a possible landmark location but no landmark encounter occurs, the particle is deleted by setting its weight to zero; afterwards the particle weights are renormalized. A complication to this implementation is that a subsequent landmark encounter only occurs if the current landmark is different than the previous one (‘landmark encounters’); to prevent the deletion of particles that correctly report a landmark at the current position but do not receive a landmark encounter signal because it is the same landmark as previously encountered, particles are deleted only if they come within the observation threshold *δ* to a possible landmark that is different than the last landmark and do not encounter it. In case all particles have been deleted, particles are resampled from a uniform distribution and their weights are equalized. As for the basic particle filter, particles are resampled whenever the effective number of particles becomes too small ${N}_{\text{eff}}=1/{\sum }_{\beta }{w}_{t,\beta }^{2} < {N}_{p}/5$. The particle filter estimate ${\hat{y}}_{t}={{\arg }}({\sum }_{\beta }{w}_{t,\beta }\exp (i{y}_{t,\beta }))$ and the circular variance $\mathrm{var}\left({y}_{t}\right)=1-\left|{\sum }_{\beta }{w}_{t,\beta }\exp \left(i{y}_{t,\beta }\right)\right|$ are also calculated in an identical way.

#### Analysis of location disambiguation in output layer

The timing and accuracy of location disambiguation in Extended Data Fig. 5 was calculated in the following way: we first constructed the trajectory of the ‘alternative location hypothesis,’ corresponding to the location estimates of a model animal that made the wrong location disambiguation at the first landmark encounter, but otherwise updated its location by the correct velocity. This trajectory is shifted relative to the true trajectory by a constant distance equal to the distance between the two landmarks. At each point in time, we then identified the two neurons in the output population whose preferred locations were closest to that of the true and alternative trajectory, respectively; the activation of these neurons roughly corresponded to the height of the activation bump corresponding to the true and alternative location hypothesis as seen in Extended Data Fig. 5c,d,h. The disambiguation time was defined as the earliest time after which either the true or alternative location bump height fell below a threshold of 0.1 and stayed beyond that threshold until the end of the trial. To determine the accuracy of location disambiguation the network estimate at the last landmark interaction was analyzed. If this network estimate was closer to the true than to the wrong landmark location the trial was categorized as a correct trial, otherwise it was categorized as an incorrect trial.

#### State space analysis

We performed PCA on the hidden neuron states from training trials to obtain the top three principal directions. We then projected network states obtained from the distribution of testing trials 2 or 3 (Supplementary Information) onto these principal directions. The resulting reduced-dimension versions of the hidden neuron states from testing trials are shown in Fig. 2 and Extended Data Figs. 5 and 10.

#### Correlation dimension

To calculate the correlation dimension for the ANN and RSC activity, we first performed linear dimensionality reduction (PCA) on hidden layer activations from the training trials, retaining 20 principal components. For RSC data, rates were first low-pass filtered at 0.5 Hz. In the 20-dimensional space, we randomly picked 1,000 base points (500 for RSC). From each of these base points, we estimated how the number of neighbors in a ball of radius *R* scales with R. The minimum ball radius was determined such that the logarithm of the number of neighbors averaged over base points was near 1. The maximum radius was set to ten times the minimum radius, and intermediate values for the radius were spaced equally on a log scale. The slope of the linear part of the relationship between the logarithm of number of neighbors versus ball radius determined the fractal dimension

### Reporting summary

Further information on research design is available in the Nature Portfolio Reporting Summary linked to this article.

## Online content

Any methods, additional references, Nature Portfolio reporting summaries, source data, extended data, supplementary information, acknowledgements, peer review information; details of author contributions and competing interests; and statements of data and code availability are available at 10.1038/s41593-025-01944-z.

## Supplementary information

Reporting Summary

## Data Availability

The experimental data of this study are available via Figshare at 10.6084/m9.figshare.27890997 (ref. ^59^).

## Code availability

Code for training ANNs is available at: https://github.com/jvoigts/Voigts_Kanitscheider_et_al_2024.

## References

[1] Vyas, S., Golub, M. D., Sussillo, D. & Shenoy, K. V. Computation through neural population dynamics. *Annu. Rev. Neurosci.* **43**, 249–275 (2020). 32640928 10.1146/annurev-neuro-092619-094115 PMC7402639

[2] Sarafyazd, M. & Jazayeri, M. Hierarchical reasoning by neural circuits in the frontal cortex. *Science* **364**, eaav8911 (2019). 31097640 10.1126/science.aav8911

[3] Mante, V., Sussillo, D., Shenoy, K. V. & Newsome, W. T. Context-dependent computation by recurrent dynamics in prefrontal cortex. *Nature* **503**, 78–84 (2013). 24201281 10.1038/nature12742 PMC4121670

[4] Smith, R. C. & Cheeseman, P. On the representation and estimation of spatial uncertainty. *Int. J. Robot. Res.* **5**, 56–68 (1986).

[5] Cho, J. & Sharp, P. E. Head direction, place, and movement correlates for cells in the rat retrosplenial cortex. *Behav. Neurosci.* **115**, 3–25 (2001). 11256450 10.1037/0735-7044.115.1.3

[6] Alexander, A. S. & Nitz, D. A. Retrosplenial cortex maps the conjunction of internal and external spaces. *Nat. Neurosci.* **18**, 1143–1151 (2015). 26147532 10.1038/nn.4058

[7] Voigts, J. & Harnett, M. T. Somatic and dendritic encoding of spatial variables in retrosplenial cortex differs during 2D navigation. *Neuron* **105**, 237–245.e4 (2020). 31759808 10.1016/j.neuron.2019.10.016 PMC6981016

[8] Mao, D., Kandler, S., McNaughton, B. L. & Bonin, V. Sparse orthogonal population representation of spatial context in the retrosplenial cortex. *Nat. Commun.* **8**, 243 (2017). 28811461 10.1038/s41467-017-00180-9 PMC5557927

[9] Hattori, R., Danskin, B., Babic, Z., Mlynaryk, N. & Komiyama, T. Area-specificity and plasticity of history-dependent value coding during learning. *Cell* **177**, 1858–1872.e15 (2019). 31080067 10.1016/j.cell.2019.04.027 PMC6663310

[10] Murakami, T., Yoshida, T., Matsui, T. & Ohki, K. Wide-field Ca2^+^ imaging reveals visually evoked activity in the retrosplenial area. *Front. Mol. Neurosci.* **8**, 20 (2015). 26106292 10.3389/fnmol.2015.00020 PMC4458613

[11] Fischer, L. F., Mojica Soto-Albors, R., Buck, F. & Harnett, M. T. Representation of visual landmarks in retrosplenial cortex. *eLife* **9**, e51458 (2020). 32154781 10.7554/eLife.51458 PMC7064342

[12] Voigts, J., Newman, J. P., Wilson, M. A. & Harnett, M. T. An easy-to-assemble, robust, and lightweight drive implant for chronic tetrode recordings in freely moving animals. *J. Neural Eng.* **17**, 026044 (2020). 32074511 10.1088/1741-2552/ab77f9 PMC8878001

[13] Burak, Y. & Fiete, I. R. Accurate path integration in continuous attractor network models of grid cells. *PLoS Comput. Biol.* **5**, e1000291 (2009). 19229307 10.1371/journal.pcbi.1000291 PMC2632741

[14] Samsonovich, A. & McNaughton, B. L. Path integration and cognitive mapping in a continuous attractor neural network model. *J. Neurosci.* **17**, 5900–5920 (1997). 9221787 10.1523/JNEUROSCI.17-15-05900.1997 PMC6573219

[15] Widloski, J. & Fiete, I. R. A model of grid cell development through spatial exploration and spike time-dependent plasticity. *Neuron* **83**, 481–495 (2014). 25033187 10.1016/j.neuron.2014.06.018

[16] Hardcastle, K., Ganguli, S. & Giocomo, L. M. Environmental boundaries as an error correction mechanism for grid cells. *Neuron* **86**, 827–839 (2015). 25892299 10.1016/j.neuron.2015.03.039

[17] Hollup, S. A., Molden, S., Donnett, J. G., Moser, M.-B. & Moser, E. I. Accumulation of hippocampal place fields at the goal location in an annular watermaze task. *J. Neurosci.* **21**, 1635–1644 (2001). 11222654 10.1523/JNEUROSCI.21-05-01635.2001 PMC6762966

[18] Lee, I., Griffin, A. L., Zilli, E. A., Eichenbaum, H. & Hasselmo, M. E. Gradual translocation of spatial correlates of neuronal firing in the hippocampus toward prospective reward locations. *Neuron* **51**, 639–650 (2006). 16950161 10.1016/j.neuron.2006.06.033

[19] Nieh, E. H. et al. Geometry of abstract learned knowledge in the hippocampus. *Nature* **595**, 80–84 (2021). 34135512 10.1038/s41586-021-03652-7 PMC9549979

[20] Remington, E. D., Narain, D., Hosseini, E. A. & Jazayeri, M. Flexible sensorimotor computations through rapid reconfiguration of cortical dynamics. *Neuron* **98**, 1005–1019.e5 (2018). 29879384 10.1016/j.neuron.2018.05.020 PMC6009852

[21] Finkelstein, A. et al. Attractor dynamics gate cortical information flow during decision-making. *Nat. Neurosci.* **24**, 843–850 (2021). 33875892 10.1038/s41593-021-00840-6

[22] Sleezer, B. J., Castagno, M. D. & Hayden, B. Y. Rule Encoding in Orbitofrontal Cortex and Striatum Guides Selection. *J. Neurosci.* **36**, 11223–11237 (2016). 27807165 10.1523/JNEUROSCI.1766-16.2016 PMC5148240

[23] Panichello, M. F. & Buschman, T. J. Shared mechanisms underlie the control of working memory and attention. *Nature* **592**, 601–605 (2021). 33790467 10.1038/s41586-021-03390-w PMC8223505

[24] Scott, B. B. et al. Fronto-parietal cortical circuits encode accumulated evidence with a diversity of timescales. *Neuron* **95**, 385–398.e5 (2017). 28669543 10.1016/j.neuron.2017.06.013 PMC9453285

[25] Harvey, C. D., Coen, P. & Tank, D. W. Choice-specific sequences in parietal cortex during a virtual-navigation decision task. *Nature* **484**, 62–68 (2012). 22419153 10.1038/nature10918 PMC3321074

[26] Yang, T. & Shadlen, M. N. Probabilistic reasoning by neurons. *Nature* **447**, 1075–1080 (2007). 17546027 10.1038/nature05852

[27] Odoemene, O., Nguyen, H. & Churchland, A. K. Visual evidence accumulation behavior in unrestrained mice. *J. Neurosci.* **38**, 10142–10155 (2018). 10.1523/JNEUROSCI.3478-17.2018 PMC6246883 30322902

[28] Xue, C., Kramer, L. E. & Cohen, M. R. Dynamic task-belief is an integral part of decision-making. *Neuron* **110**, 2503–2511.e3 (2022). 35700735 10.1016/j.neuron.2022.05.010 PMC9357195

[29] Guo, W., Zhang, J. J. & Wilson, M. A. Latent learning drives sleep-dependent plasticity in distinct CA1 subpopulations. *Cell Rep.* **43**, 115028 (2024). 39612242 10.1016/j.celrep.2024.115028

[30] Yoon, K. et al. Specific evidence of low-dimensional continuous attractor dynamics in grid cells. *Nat. Neurosci.* **16**, 1077–1084 (2013). 23852111 10.1038/nn.3450 PMC3797513

[31] Gardner, R. J. et al. Toroidal topology of population activity in grid cells. *Nature* **602**, 123–128 (2022). 35022611 10.1038/s41586-021-04268-7 PMC8810387

[32] McKenzie, S. et al. Preexisting hippocampal network dynamics constrain optogenetically induced place fields. *Neuron* **109**, 1040–1054.e7 (2021). 33539763 10.1016/j.neuron.2021.01.011 PMC8095399

[33] Banerjee, A. et al. Value-guided remapping of sensory cortex by lateral orbitofrontal cortex. *Nature* **585**, 245–250 (2020). 32884146 10.1038/s41586-020-2704-z

[34] Stachenfeld, K. L., Botvinick, M. M. & Gershman, S. J. The hippocampus as a predictive map. *Nat. Neurosci.* **20**, 1643–1653 (2017). 28967910 10.1038/nn.4650

[35] Inagaki, H. K., Fontolan, L., Romani, S. & Svoboda, K. Discrete attractor dynamics underlying selective persistent activity in frontal cortex. *Nature* **566**, 212–217 (2019). 30728503 10.1038/s41586-019-0919-7

[36] Uria, B. et al. A model of egocentric to allocentric understanding in mammalian brains. Preprint at *bioRxiv*10.1101/2020.11.11.378141 (2020).

[37] Yamins, D. L. K. et al. Performance-optimized hierarchical models predict neural responses in higher visual cortex. *Proc. Natl Acad. Sci. USA* **111**, 8619–8624 (2014). 24812127 10.1073/pnas.1403112111 PMC4060707

[38] Gallego, J. A. et al. Cortical population activity within a preserved neural manifold underlies multiple motor behaviors. *Nat. Commun.* **9**, 4233 (2018). 30315158 10.1038/s41467-018-06560-z PMC6185944

[39] Ma, W. J., Beck, J. M., Latham, P. E. & Pouget, A. Bayesian inference with probabilistic population codes. *Nat. Neurosci.* **9**, 1432–1438 (2006). 17057707 10.1038/nn1790

[40] Echeveste, R., Aitchison, L., Hennequin, G. & Lengyel, M. Cortical-like dynamics in recurrent circuits optimized for sampling-based probabilistic inference. *Nat. Neurosci.* **23**, 1138–1149 (2020). 32778794 10.1038/s41593-020-0671-1 PMC7610392

[41] Lu, K., Grover, A., Abbeel, P. & Mordatch, I. Pretrained transformers as universal computation engines. Preprint at https://arxiv.org/abs/2103.05247v2 (2021).

[42] Kirkpatrick, J. et al. Overcoming catastrophic forgetting in neural networks. *Proc. Natl Acad. Sci. USA* **114**, 3521–3526 (2017). 28292907 10.1073/pnas.1611835114 PMC5380101

[43] Rigotti, M. et al. The importance of mixed selectivity in complex cognitive tasks. *Nature* **497**, 585–590 (2013). 23685452 10.1038/nature12160 PMC4412347

[44] Fusi, S., Miller, E. K. & Rigotti, M. Why neurons mix: high dimensionality for higher cognition. *Curr. Opin. Neurobiol.* **37**, 66–74 (2016). 26851755 10.1016/j.conb.2016.01.010

[45] Newman, J. P. et al. Twister3: a simple and fast microwire twister. *J. Neural Eng.*10.1088/1741-2552/ab77fa (2020). 32074512 10.1088/1741-2552/ab77fa PMC8879418

[46] Siegle, J. H. et al. Open Ephys: an open-source, plugin-based platform for multichannel electrophysiology. *J. Neural Eng.* **14**, 045003 (2017). 28169219 10.1088/1741-2552/aa5eea

[47] & Newman, J. P. et al. ONIX: a unified open-source platform for multimodal neural recording and perturbation during naturalistic behavior. *Nat. Methods* **22**, 187–192 (2025). 39528678 10.1038/s41592-024-02521-1 PMC11725498

[48] Lopes, G. et al. Bonsai: an event-based framework for processing and controlling data streams. *Front. Neuroinform.* **9**, 7 (2015). 25904861 10.3389/fninf.2015.00007 PMC4389726

[49] Chung, J. E. et al. A fully automated approach to spike sorting. *Neuron* **95**, 1381–1394.e6 (2017). 28910621 10.1016/j.neuron.2017.08.030 PMC5743236

[50] Newman, J. et al. jonnew/Oat: Oat version 1.0. *Zenodo*10.5281/zenodo.1098579 (2017).

[51] Tibshirani, R. Regression shrinkage and selection via the lasso. *J. R. Stat. Soc. Ser. B Methodol.* **58**, 267–288 (1996).

[52] Eisen, M. B., Spellman, P. T., Brown, P. O. & Botstein, D. Cluster analysis and display of genome-wide expression patterns. *Proc. Natl Acad. Sci. USA* **95**, 14863–14868 (1998). 9843981 10.1073/pnas.95.25.14863 PMC24541

[53] Tenenbaum, J. B., de Silva, V. & Langford, J. C. A global geometric framework for nonlinear dimensionality reduction. *Science* **290**, 2319–2323 (2000). 11125149 10.1126/science.290.5500.2319

[54] Van Der Maaten, L., Postma, E. & Van den Herik, J. Dimensionality reduction: a comparative. *J. Mach. Learn Res.* **10**, 13 (2009).

[55] Kingma, D. P. & Ba, J. Adam: a method for stochastic optimization. Preprint at https://arxiv.org/abs/1412.6980v9 (2017).

[56] Widloski, J. & Fiete, I. R. Inferring circuit mechanisms from sparse neural recording and global perturbation in grid cells. *eLife* **7**, e33503 (2018). 29985132 10.7554/eLife.33503 PMC6078497

[57] Welinder, P. E., Burak, Y. & Fiete, I. R. Grid cells: the position code, neural network models of activity, and the problem of learning. *Hippocampus* **18**, 1283–1300 (2008). 19021263 10.1002/hipo.20519

[58] Thrun, S., Burgard, W. & Fox, D. *Probabilistic Robotics* (MIT, 2005).

[59] Voigts, J. Spatial reasoning via recurrent neural dynamics in mouse retrosplenial cortex. *Figshare*10.6084/m9.figshare.27890997 (2025). 10.1038/s41593-025-01944-z PMC12148932 40481228

[60] Kropff, E., Carmichael, J. E., Moser, M.-B. & Moser, E. I. Speed cells in the medial entorhinal cortex. *Nature* **523**, 419–424 (2015). 26176924 10.1038/nature14622

[61] Jeewajee, A., Barry, C., O’Keefe, J. & Burgess, N. Grid cells and theta as oscillatory interference: electrophysiological data from freely moving rats. *Hippocampus* **18**, 1175–1185 (2008). 19021251 10.1002/hipo.20510 PMC3173868

[62] Fiser, J., Berkes, P., Orbán, G. & Lengyel, M. Statistically optimal perception and learning: from behavior to neural representations. *Trends Cogn. Sci.* **14**, 119–130 (2010). 20153683 10.1016/j.tics.2010.01.003 PMC2939867

[63] Zhang, K. Representation of spatial orientation by the intrinsic dynamics of the head-direction cell ensemble: a theory. *J. Neurosci.* **16**, 2112–2126 (1996). 8604055 10.1523/JNEUROSCI.16-06-02112.1996 PMC6578512

[64] Tsodyks, M. & Sejnowski, T. Associative memory and hippocampal place cells. *Int. J. Neural Syst.* **6**, 81–86 (1995).

[65] Meshulam, L., Gauthier, J. L., Brody, C. D., Tank, D. W. & Bialek, W. Collective behavior of place and non-place neurons in the hippocampal network. *Neuron* **96**, 1178–1191.e4 (2017). 29154129 10.1016/j.neuron.2017.10.027 PMC5720931

[66] Stefanovska, A., Strle, S. & Krošelj, P. On the overestimation of the correlation dimension. *Phys. Lett. A* **235**, 24–30 (1997).

[67] Gothard, K. M., Skaggs, W. E. & McNaughton, B. L. Dynamics of mismatch correction in the hippocampal ensemble code for space: interaction between path integration and environmental cues. *J. Neurosci.* **16**, 8027–8040 (1996). 8987829 10.1523/JNEUROSCI.16-24-08027.1996 PMC6579211

[68] Recanatesi, S., Ocker, G., Buice, M. & Shea-Brown, E. Dimensionality in recurrent spiking networks: global trends in activity and local origins in connectivity. *PLoS Comput. Biol.* **15**, e1006446 (2019). 31299044 10.1371/journal.pcbi.1006446 PMC6655892

